# Reciprocal Loss of CArG-Boxes and Auxin Response Elements Drives Expression Divergence of MPF2-Like MADS-Box Genes Controlling Calyx Inflation

**DOI:** 10.1371/journal.pone.0042781

**Published:** 2012-08-10

**Authors:** Muhammad Ramzan Khan, Jinyong Hu, Ghulam Muhammad Ali

**Affiliations:** 1 Max-Planck-Institute for Plant Breeding Research, Department of Molecular Plant Genetics, Cologne, Germany; 2 National Institute for Genomics and Advanced Biotechnology, National Agricultural Research Centre, Islamabad, Pakistan; Michigan State University, United States of America

## Abstract

Expression divergence is thought to be a hallmark of functional diversification between homologs post duplication. Modification in regulatory elements has been invoked to explain expression divergence after duplication for several MADS-box genes, however, verification of reciprocal loss of *cis*-regulatory elements is lacking in plants. Here, we report that the evolution of *MPF2-like* genes has entailed degenerative mutations in a core promoter CArG-box and an auxin response factor (ARF) binding element in the large 1^st^ intron in the coding region. Previously, *MPF2-like* genes were duplicated into *MPF2-like-A* and *-B* through genome duplication in *Withania* and *Tubocapsicum* (Withaninae). The calyx of *Withania* grows exorbitantly after pollination unlike *Tubocapsicum*, where it degenerates. Besides inflated calyx syndrome formation, MPF2-like transcription factors are implicated in functions both during the vegetative and reproductive development as well as in phase transition. *MPF2-like-A* of *Withania* (*WSA206*) is strongly expressed in sepals, while *MPF2-like-B* (*WSB206*) is not. Interestingly, their combined expression patterns seem to replicate the pattern of their closely related hypothetical progenitors from *Vassobia* and *Physalis*. Using phylogenetic shadowing, site-directed mutagenesis and motif swapping, we could show that the loss of a conserved CArG-box in *MPF2-like-B* of *Withania* is responsible for impeding its expression in sepals. Conversely, loss of an ARE in *MPF2-like-A* relaxed the constraint on expression in sepals. Thus, the ARE is an active suppressor of *MPF2-like* gene expression in sepals, which in contrast is activated via the CArG-box. The observed expression divergence in *MPF2-like* genes due to reciprocal loss of *cis*-regulatory elements has added to genetic and phenotypic variations in the Withaninae and enhanced the potential of natural selection for the adaptive evolution of ICS. Moreover, these results provide insight into the interplay of floral developmental and hormonal pathways during ICS development and add to the understanding of the importance of polyploidy in plants.

## Introduction

Gene or genome duplications and subsequent expression divergence due to modifications in regulatory elements create morphogenetic novelties [1]. However, our current understanding of the selective regulatory changes arising post-duplication is preliminary and fragmented. Among transcription factors families, the MADS family has greatly expanded in plants by a series of duplications that enabled the genes to diversify in structure and function [2]. Alterations in the expression profiles of MADS-domain proteins subsequent to regulatory changes are closely linked to the origin of developmental and morphological novelties, most conspicuously the floral organs of angiosperms. The inflated calyx syndrome (ICS) is a spectacular floral morphological novelty exhibited by a few genera of Solanaceae [3]. After pollination, *Withania* and *Physalis* sepals resume growth and give rise to a balloon-like structure, i.e. ICS or “Chinese lantern” encapsulating the berry [4]. The genus *Withania* consists of 11 species, which are worldwide in distribution. It displays a variety of inflated calyces ranging from the half open balloons of *W. aristata* and *W. frutescens* containing needle-like and teeth-like projections, respectively, to open fleshy “lanterns” in *W. riebeckii*, and completely closed semi-succulent papery lanterns in *W. somnifera* and *W. coagulans* [5]. By sharp contrast, *Tubocapsicum* features only a rudimentary calyx and is considered to be an “evolutionary loss mutant” of the ICS. This genus consists of two species, mainly endemic to humid regions of Eastern Asia. Phylogenetically, *Withania* and *Tubocapsicum* are sister genera to each other and are placed in the sub-tribe Withaninae along with 7 other genera [4].

The unique and single copy MADS-box gene *MPF2*– an ortholog of the potato gene *STMADS16* - controls ICS formation in *Physalis* [6]. Previously, changes in its promoter region were shown to account for the expression of *MPF2* in floral organs. However, in the tetraploid Withaninae, probably due to allotetraploidization, *MPF2* is duplicated into *MPF2-like-A* and *-B* genes, of which only the former controls ICS formation in *Withania*. This functional divergence is a consequence of differences in gene expression potentially mediated by their divergent promoters [5]. It is generally believed that variations in gene expression are an important source of phenotypic diversity. The identification of regulatory changes underlying specific expression differences, however, is more difficult and hitherto, little progress has been made in connecting expression divergence with regulatory sequence variation. In *Withania*, the evidence for divergent expression of *MPF2-like* duplicates suggests an important control exerted by *cis*-regulatory regions, and prompted us to assess, if divergent elements in the promoter and intronic regions are responsible. Indeed, a conservation-based approach revealed that *MPF2-like-A* promoters lack an ARF (auxin response factor) binding site in the large 1^st^ intron in the coding region whereas its homolog *MPF2-like-B* is devoid of a CArG-box near the transcriptional start site. This CArG-box is responsible for expression in sepals, which is essential for ICS formation. By contrast, the ARF binding element in the 1^st^ large intron suppresses the expression in flower/calyx as evident from site-directed mutagenesis using reporter genes driven by *MPF2-like cis*-elements. Based on structural and functional characterization of *MPF2-like cis*-regulatory elements, we posit that adaptive evolution of *MPF2-like* genes was achieved at least in part by degenerative mutations in the core promoter CArG-box and the ARF binding site in the large1^st^ intron of the coding region. Moreover, our data provide insight into auxin signaling as an input pathway for *MPF2-like* genes.

## Results

### *Withania* Calyx Cells Grow after Pollination

**Figure 1:**
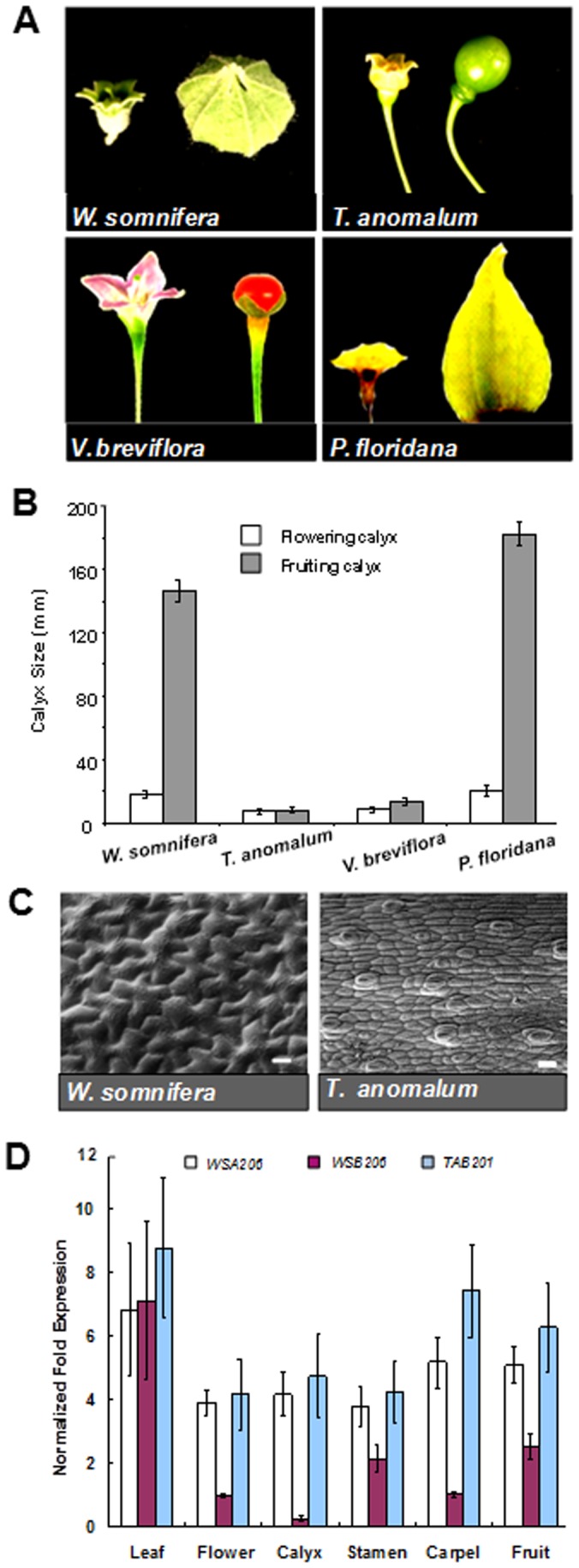
Morphological diversity of inflated calyx and expression of *MPF2-like* genes. A ) Photographs exhibiting diversity in the flowering and fruiting calyces of *Withania*, *Tubocapsicum*, *Vassobia* and *Physalis*. **B**) Graph showing variations in flowering and fruiting calyces of *Withania*, *Tubocapsicum*, *Vassobia* and *Physalis*. Length and width of 10 calyces at different position of flowering and fruiting calyces were measured using a Vernier scale and size of calyx was calculated. Different accessions of Solanaceous plants are indicated on horizontal axis. Error bars indicate the standard deviations of the mean. **C**) Scanning electron microscopy of *Withania* exhibits differences in the growth patterns of *Withania* and *Tubocapsicum* calyx epidermal cells surrounding berry. Note the increase and lobation of *Withania* cells in comparison with *Tubocapsicum*. Bars correspond to 20 mm. **D**) Expression analysis of *MPF2-like* genes. The RNAs isolated from leaves, flower buds, sepals, stamens, carpels and siliques of *Withania somnifera* and *Tubocapsicum anomalum* were subjected to real-time RT-PCR analysis with gene specific primer pairs. The columns show the expression of *MPF2-like-A* of *Withania* (*WSA206*; white), *MPF2-like-B* of *Withania* (*WSB206*; reddish brown) and *MPF2-like-B* of *Tubocapsicum* (*TAB201*; light blue). The values given are relative expression based on three independent experiments normalized with respect to 18 S *rRNA*. Error bars indicate the standard deviation.

*Withania* and *Tubocapsicum* though display contrasting phenotypes with regard to calyx inflation, are close relatives of *Physalis* and sister genera to each other. Therefore, in order to observe to what extent the calyx of *Withania* and *Tubocapsicum* increases in size after fertilization, we compared their flowering and fruiting calyces, i.e. before and after fertilization (Fig. 1A). The measurements revealed an exorbitant increase (4 to 5 times) in the fruiting calyx of *Withania*. By sharp contrast, the fruiting calyx of *Tubocapsicum* was even smaller than the flowering calyx showing its degeneration after pollination (Fig. 1B). By analogy, *Vassobia* a closely related genus to *Tubocapsicum* also exhibited no post-fertilization calyx inflation. These data suggest that after pollination or during fruit development both the flowering and fruiting calyces of *Withania* can change in size and architecture. Figure 1 C shows that post anthesis when fruits are developing; unlike in *Withania* where cells increase in size and become lobed, *Tubocapsicum* sepal cells are slightly stretched out but neither larger nor lobed. From this data we can infer that only in the *Withania* calyx the morphology of cells changes post fertilization.

### *MPF2-like-B* of *Withania* (*WSB206*) is not Expressed in the Calyx

RNA was isolated from leaf and floral tissues of *Withania* and *Tubocapsicum*, and subjected to quantitative real-time RT-PCR (see Materials and Methods). Figure 1D demonstrates that all the *MPF2-like* genes are strongly expressed in leaves. The transcript signals of *Withania MPF2-like-A* (*WSA206*) and *Tubocapsicum MPF2-like-B* (*TAB201*) are detectable in all the floral organs, i.e. calyx, stamen, carpel and fruit. Conversely, the precocious expression of *Withania MPF2-like-B* (*WSB206*) is weak in stamens, carpels and fruits. Surprisingly, *WSB206* transcript is absent in sepals, which precludes its role in ICS formation. Hence, the expression of the Withaninae *MPF2-like* gene duplicates *MPF2-like-A* and *–B* have diverged. These genes have originated presumably after genome merger (allotetraploidization) in the Withaninae. Which gene was contributed by which progenitor would be exciting to elucidate. Therefore, in order to get an indication about their hypothetical progenitors, the phylogeny of Physaleae (a tribe in Solanaceae that includes Physalinae, Withaninae, Iochrominae etc.) was established.

### Combined Expression Patterns of *MPF2-like* Duplicates of *Withania* Replicate their Hypothetical Progenitor

**Figure 2:**
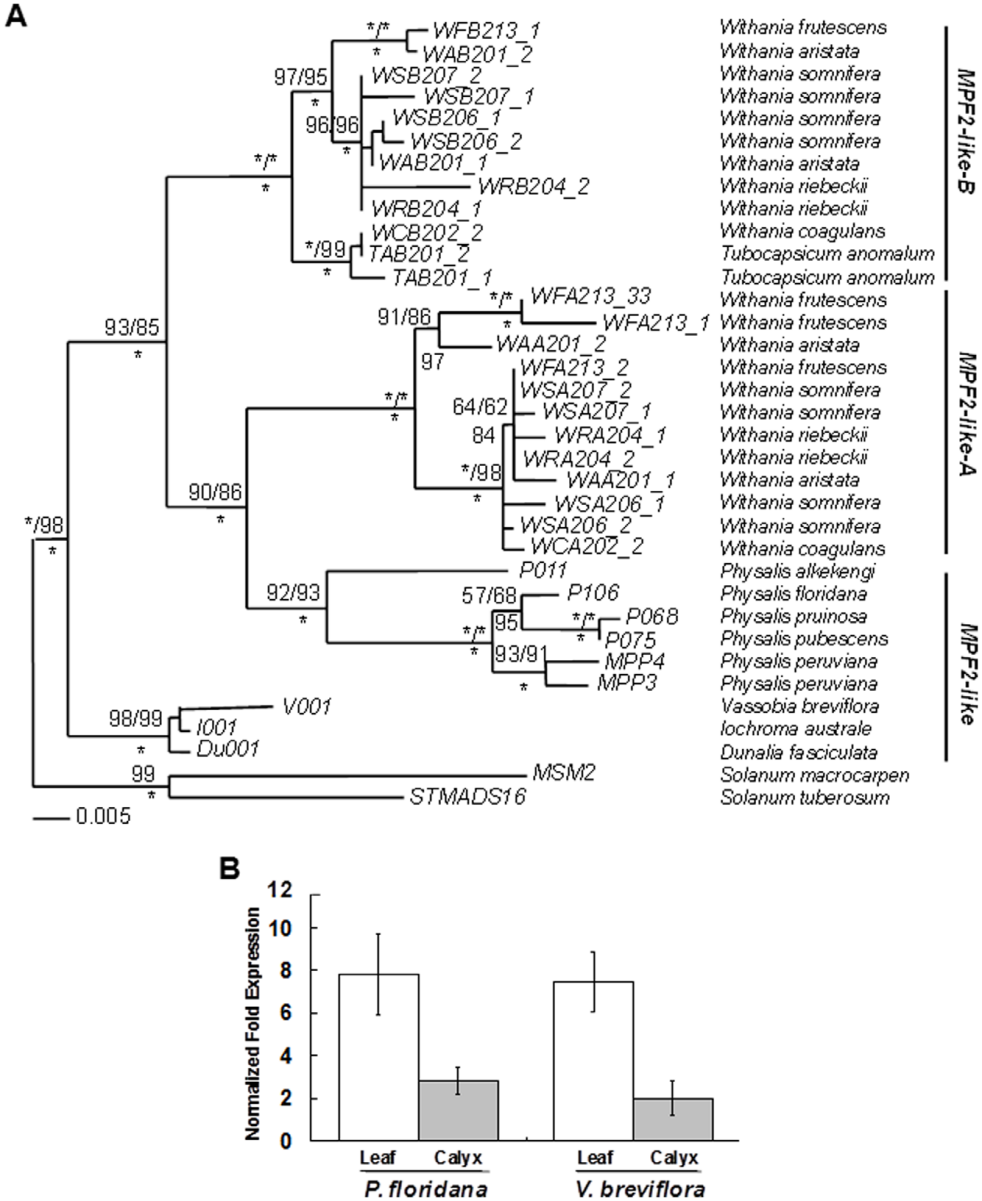
Phylogenetic reconstruction of *MPF2-like* genes and expression patterns of their progenitors. A ) A maximum likelihood tree of *MPF2-like* sequences from various Solanaceous species is established using *STMADS16* as an out-group. The ML tree reconstruction is carried out with PAUP 4.0b10. The robustness of the tree structure is evaluated by 1000 replicates of bootstrap searches using maximum parsimony (MP) and maximum likelihood (ML) in PAUP and Bayesian posterior probability is indicated as *. The multiple sequences for a gene are also indicated. **B**) The expression patterns of *MPF2-like* duplicates replicate their subsumed progenitors. RNA isolated from leaves and sepals of *Physalis* and *Vassobia* was subjected to real-time RT-PCR analysis. The values given for leaf (empty bar) and calyx (light grey bar) represent the relative expression normalized with respect to 18 S *rRNA*. Error bars indicate the standard deviation.

We constructed a maximum likelihood phylogeny of *MPF2-like* genes using the PAUP4.0B10 software (Fig. 2A). Phylogenetic analysis clearly differentiated the *MPF2-like* genes into three distinct clades designated as *MPF2-like-A*, *MPF2-like-B* and *MPF2-like*. Multiple copies of the genes found in *Withania* clustered into their respective gene clades, i.e. *MPF2-like-A* and *–B*, respectively. *MPF2* from *Physalis* was found in a sister position with respect to the *MPF2-like-A* subclade, while Iochrominae genes (*V001*, *1001* and *Du001*) occupied a basal position to both the *MPF2-like-A* and *MPF2-like-B* clades. These phylogenetic analyses allow us to speculate that for the allotetraploidization of *MPF2-like* genes of Withaninae, *Physalis* might be the contributor of *MPF2-like-A* and Iochrominae of *MPF-like-B,* respectively. This inference is supported by their protein sequence similarities. The pairwise alignment showed that V001 and WSB206 (MPF2-like-B) are 98% similar. Therefore a closely related gene might have been the progenitor of the MPF2-like-B protein. Interestingly, MPF2 is 87% identical with both WSA206 (MPF2-like-A) and WSB206 (MPF2-like-B). As both WSA206 (MPF2-like-A) and MPF2 play an important role in calyx inflation, thus, MPF2 is clearly closely related to MPF2-like-A (WSA206) functionally [5] (Fig. S1).

One of the characteristics of divergence after duplication is that combined expression patterns of the duplicates should be reminiscent of their progenitors. Therefore expression patterns of *Physalis* and *Vassobia* were studied in leaf and calyx tissues (Fig. 2B). Quantitative real-time RT-PCR revealed that both *MPF2* and *V001* were strongly expressed in leaves like the Withaninae duplicates. Although weaker, the transcript signals of both genes were also detectable in the calyx [4]. Thus, combined expression of the duplicates (*Withania MPF2-like-A* and *-B*) seems to be equivalent to the expression of unduplicated homologs in the closely related species of *Physalis* and *Vassobia*. Clearly, these observations support the occurrence of expression divergence after the genome duplication in Withaninae and allude towards their hypothetical diploid progenitors.

Previously, promoter mutations were shown to be implicated in heterotopic expression of *MPF2* of *Physalis* in floral organs in comparison with *STMADS16* of *Solanum*, which is strictly vegetative in expression [6]. In most of the cases, expression divergence in paralogs observed after duplication, is because of modifications in *cis*-regulatory elements [7], [8]. Therefore, we decided to analyze the promoters and large 1^st^ intron sequences of *MPF2-like* genes with various bioinformatic tools to identify the putative *cis*-elements responsible for their divergent expression.

### Phylogenetic Shadowing Reveals Reciprocal Loss of *cis*-Regulatory Elements in *MPF2-like-A* and *-B* Genes Post Duplication

**Figure 3:**
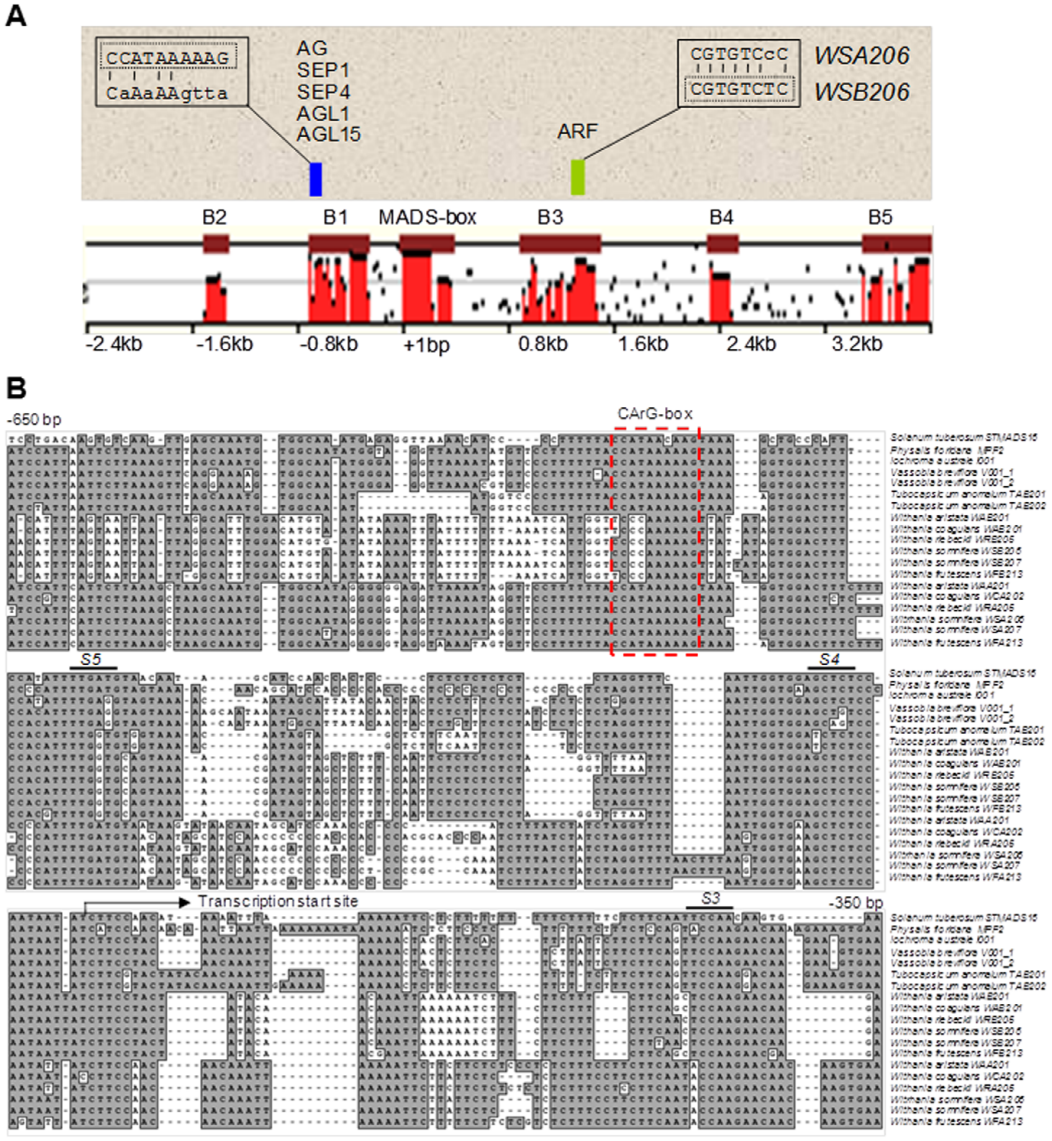
Bioinformatics analysis and phylogenetic shadowing of *MPF2-like* regulatory elements. A ) Mulan analysis of *MPF2-like* promoters and large 1^st^ introns including MADS-box. Two conserved blocks (B1, B2) are identified in the promoter region and three (B3–B5) in the large1^st^ intron in the coding region. The first conserved block (B1) in the promoter region contains a CArG-box, which is present in all the sequence except *MPF2-like-B* of *Withania* (*WSB206*) and *STMADS16*. This CArG-box is a binding site for MADS-domain proteins such as AG, SEP1, SEP4, AGL1 and AGL15. The wild type sequence (in the dotted box) of this CArG-box is shown for *WSA206* gene and mutated one for *WSB206*. In the large 1^st^ intron first conserved block (B3; Fig. S4) contains an ARF binding site at variable positions in all the *MPF2-like* sequences except *MPF2-like-A* of *Withania* (*WSA206*). The normal ARF binding site (in the dotted box) is shown for *WSB206*, while the mutated one for *WSA206* is also depicted. A rough scale is indicated. The position of translational start site is marked as +1. **B**) ClustalW multiple alignment of the core promoter conserved block (B1) of *MPF2-like* promoter sequences (**−**350 bp to **−**650 bp). Five conserved sequences stretches are identified (Fig. S3) and called as *shadows 1* to *5* (*S1* to *S5*). Only *S3* to *S5* are shown in this alignment. Backward arrow marks the putative transcription start site. Dotted red box encloses the conserved CArG-box sequence.

We aligned *MPF2-like* promoter sequences (more than 2 kb upstream of the translational start site) as well as sequences of the large 1^st^ intron in the coding region (more than 2 kb downstream of translational start site) from various genera. MULAN and mVISTA analyses revealed 2 highly conserved homology blocks B1 and B2 in the promoter region and 3 blocks B3, B4 and B5 in the large 1^st^ intron (Fig. 3A). The first conserved block (B1) in the promoter includes 5′ UTRs and the TSS and may be designated as the core promoter. ClustalW alignments were used to analyze, whether these conserved blocks contain functional regulatory motifs potentially responsible for divergent expression. Figure 3B demonstrates that five of these putative sequences did not contain documented transcription factor binding sites for MADS-box genes. Therefore we called them *shadows S1–S5* (Fig. S2). Sequence analysis of entire **−**2 kb *MPF2-like* promoters revealed different categories of MADS-domain binding motifs (CArG-boxes) including an N10-like (C(A/T)_8_G), CC(A/T)_6_GG, and CC(A/T)_7_G CArG motifs, (the subscript represent the number of A/T base pairs). These CArG motifs were frequently found throughout the *MPF2-like* promoter and 1^st^ intron (Table 1 and Fig. S3). A CC(A/T)_7_G type CArG-box (CCATAAAAAG) in the vicinity of the TSS was identified in all the sequences except *MPF2-like-B* of *Withania* (*WSB206*). Besides this core-promoter CArG-box, which is a reported binding site for MADS-box proteins such as AG, AGL1, SEP1, SEP4 and AGL15 in *Arabidopsis* [9], binding sites for homeodomain proteins (AtHB1, AtHB5), AGP1 and P-binding protein were identified in all the *MPF2-like-A* and *–B* promoters. Interestingly, all these latter transcription factor-binding sites are located within the 5′-UTR region, while the CArG-box is found upstream of the TSS at fairly constant distance (approximately 10 to 12 helical turns of the DNA).

**Table 1:** Specifications and positions of different *cis*-regulatory elements of the *MPF2-like* genes.

MPF2-like gene	Promoter length (bp)	Transcription start site	Position of CArG-box in promoter region	Intron length (bp)	Position of CArG-box in the large 1^st^ intron	Position of ARE
WSA206	2606	**−**320	**−**430, **−**2050, **−**2550,	2544	474, 2092	694^∶M^
WSB206	3348	**−**415	460*^M^, **−**698, **−**1754	1831	1133, 1627	430
TAB201	2308	**−**436	**−**560, 1290, **−**1557, **−**1851	3704	697, 1493, 2010, 2888, 2995, 3048	1214
MPF2	2237	**−**472	**−**580, 1488, **−**1784, **−**2091	2159	549, 855, 1429	786
STMADS16	2588	**−**459	**−**32, **−**249, **−**460*^M^, **−**937, **−**1226, **−**2147	2539	234, 314, 558, 717, 921, 1296	1187
V001_1	2056	**−**594	**−**440, **−**836, **−**1285,	3386	186, 812, 945, 2377, 2539, 2781, 2896, 3311	1344

The position of translational start site is +1. *^M^ indicates the position of lost or mutated CArG-box in *WSB206* and *STMADS16* core promoters. whereas mutated ARF binding site in the large 1^st^ intron of *WSA206* is indicated as ^⊗M^.

Remarkably, in the 1^st^ conserved block (B3) of the large 1^st^ intron in the coding region, a 200 bp highly conserved region was identified in all the *MPF2-like* sequences at variable positions. Within this 200 bp conserved stretch of DNA, ClustalW alignment revealed an auxin response factor (ARF) binding element (ARE; cgTGTCTC) to be conserved in all the sequences except *MPF2-like-A* (*WSA206*) of *Withania* (Fig. S4). This element is preferentially bound by a glutamine rich type of ARF (ARF_Q2 element) known to activate expression [10].

In conclusion, the data show the loss of an otherwise conserved core-promoter CArG-box in *MPF2-like-B* and of an ARF binding site in the large 1^st^ intron of *MPF2-like-A* from *Withania*, respectively. The reciprocal loss of *cis*-regulatory elements is a characteristic of divergence after duplication. Remarkably, both the *Physalis* and *Vassobia MPF2-like genes -* the presumed progenitors of *MPF2-like-A* and *–B* genes, respectively - contain the core-promoter CArG-box as well as the ARE in the large 1^st^ intron.

### Loss of Core-promoter CArG-box Blocks Expression of *MPF2-like-B* Genes in Sepals

**Figure 4:**
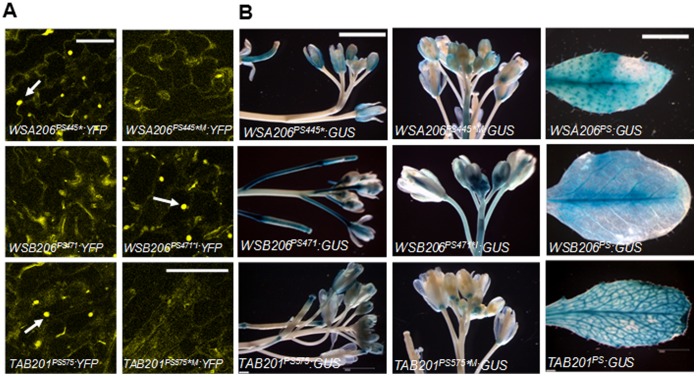
Core promoter CArG-box controls expression of *MPF2-like* genes in sepals. **A**) Confocal images of the stable expression of YFP gene in sepals of transgenic *Arabidopsis*. The YFP is fused with *MPF2-like* (*WSA206*, *WSB206* and *TAB201*) short promoters containing normal and mutated CArG-box. PS, promoter short; I, *MPF2-like* large 1^st^ intron; * CArG-box; ⊗, ARE; M, mutated; ⊗I, introduced CArG-box; I⊗, introduced ARE. Arrow points to expression of the YFP in the nucleus. The scale bar = 30 µm. **B**) Photographs showing expression of GUS reporter gene in the inflorescence of transgenic *Arabidopsis* plants transformed with *MPF2-like* short promoter:GUS constructs with normal and mutated version of core promoter CArG-box. PS, promoter short; I, *MPF2-like* large 1^st^ intron; * CArG-box; *, CArG-box; ⊗, ARE; M, mutated; *I, introduced CArG-box; I⊗, introduced ARE. The scale bar is equal to 4 mm.

Using an *in vivo* reporter assay the regulatory potential of the *MPF2-like* core promoter CArG-box was evaluated through site-directed mutagenesis and motif swapping. For this purpose chimeric promoter-reporter constructs containing normal and mutated CArG-boxes were transformed into *Arabidopsis* stably (YFP and GUS were used as reporter genes). The transformation of regulatory elements in heterologus system is now routine particularly in animals. In animal system the human regulatory elements have been successfully tested in zebra fish [11], which are evolutionarily distant relatives (450 million years: [8], [12]. It is speculated that evolutionary distance between *Arabidopsis* and *Withania* might falls within this limit as they are different at family level. Figure 4A depicts that very strong signals are detected in the nucleus of *Arabidopsis* sepals when transformed with WSA206^PS445*^:YFP, while YFP signals are diminished if the CArG-box is mutated (WSA206^PS445*M^:YFP). Remarkably, the introduction of a CArG-box in WSB206^PS471*I^:YFP plants causes a dramatic increase in YFP signal, while originally *WSB206^PS471^:YFP* lack or show little intensity in sepals. However, the native *MPF2-like-B* promoter lacking the CArG motif drives expression in sepals. Similarly, the signal strength is weaker in case of plants transformed with mutated TAB201^PS575*M^:YFP constructs, lacking an intact CArG-motif. The role of this CArG-box in controlling *MPF2-like* expression in sepals was further validated by stable promoter:GUS analysis in transgenic *Arabidopsis* (Fig. 4B). In case of WSA206^PS445*^:GUS plants the GUS expression was strongly detectable in young sepals but only weak if the CArG-box was mutated (WSA206^PS445*M^:YFP). On the other hand, introduction of a CArG-box in WSB206^PS471*^:GUS rescued the expression in young sepals, while originally WSB206^PS471*^:GUS plants showed little or no expression in the sepals. A slight decrease in GUS signals in the case of TAB201^PS575*M^:GUS (lacking the CArG-box) was also observed in comparison with TAB201^PS575*^:GUS that exhibited stronger signals. These data clearly suggest that the core-promoter CArG-box is crucial for activating expression of *MPF2-like* genes in sepals. This is supported by the lack of expression of *MPF2-like-B* (not containing a CArG-box) in the sepals of native plant (Fig. 1D).

Given our results it is paramount that effects of the entire promoter, i.e. more than **−**2 kb upstream of the translational start site, on the expression of *MPF2-like* genes should be delineated.

### *MPF2-like-B* Promoter Drives Strong GUS Expression in Transgenic *Arabidopsis* Flowers Including Sepals

**Figure 5:**
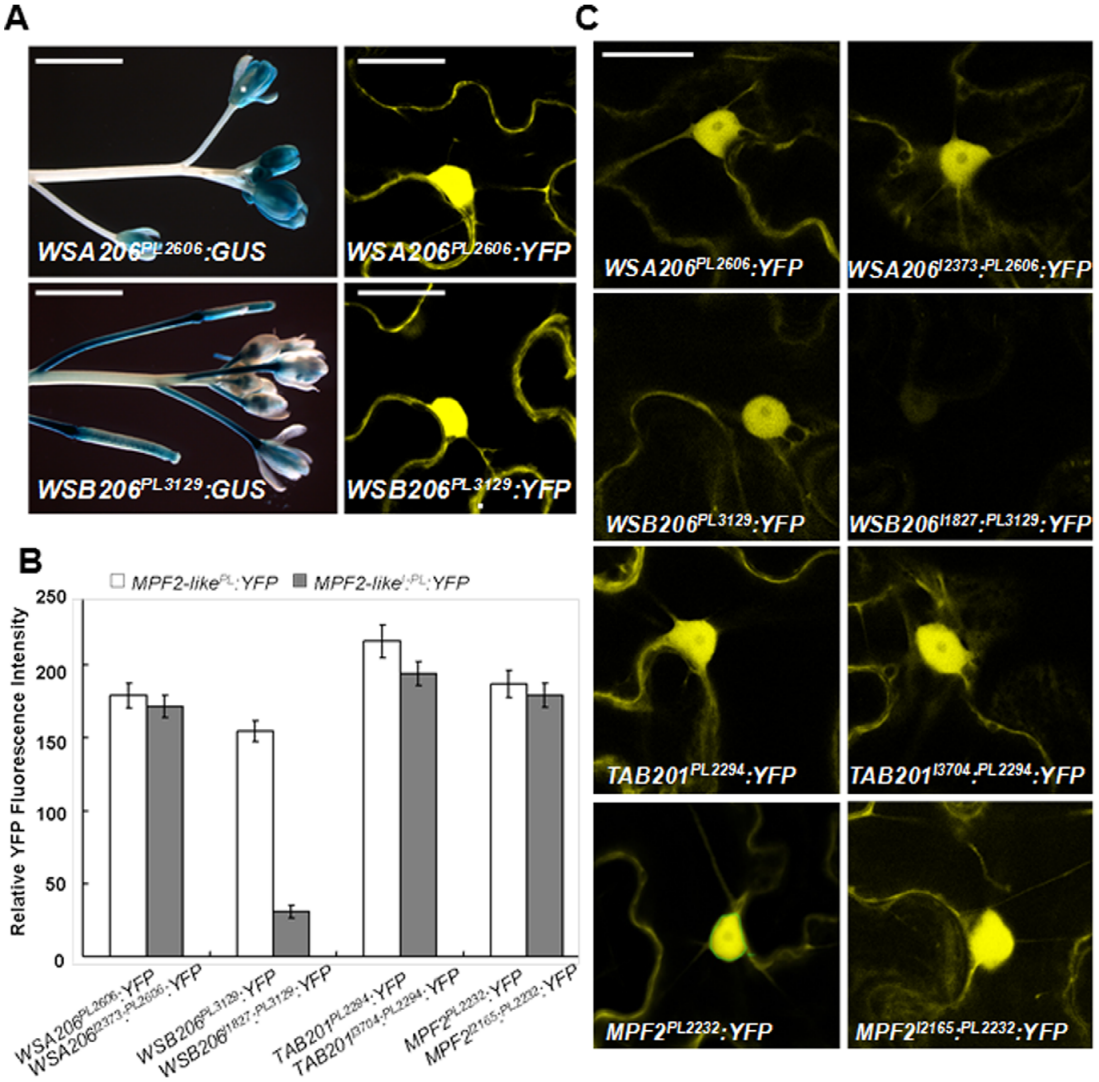
*MPF2-like* promoter activates the expression of YFP/GUS, while large 1^st^ intron suppresses it. A ) *MPF2-like* long promoter was attached with GUS and YFP reporter genes and expressed stably and transiently in *Arabidopsis* and *Nicotiana*, respectively. The GUS signals driven by *Withania MPF2-like-A* and -*B* promoter are equally strong in the inflorescence though native expression for *MPF2-like-B* is barely detectable. Similarly YFP signals are very strong in both types of promoters. The scale bar represents 4 mm and 30 µm for GUS and YFP pictures, respectively. **B**) A transient YFP expression assay was performed under the control of *MPF2-like* long promoter and complete large 1^st^ intron (*MPF2-like^PL^:YFP*; *MPF2-like^I^:^PL^:YFP*). Three days after infiltration, leaves of *N. benthamiana* were scanned under Leica LCS SP2 AOBS^R^, Confocal Laser Scanning Microscope (CLSM) for YFP signal detection. At least 10 images selected randomly to quantify the luminescence with the Leica software LCS Lite. Promoter strength was determined as the relative intensity of YFP fluorescence of nuclear area of *MPF2-like* promoter YFP constructs in comparison with nuclear YFP fluorescence intensity of a 35 S CaMV promoter YFP construct. In the case of *WSB206-like^I^:^PL^:YFP* there is a tremendous decrease in YFP signal intensity after attaching *MPF2-like* large 1^st^ intron with promoter region. The error bars represent the standard deviation. **C**) Confocal images showing YFP expression driven under *MPF2-like cis*-elements in the nuclei of the transiently transformed *Nicotiana* leaves. The scale bar represents 30 nm.

Three constructs in which GUS/YFP reporter expression is driven by the more than 2 kb long promoter of *WSA206*, *WSB206,* and *TAB201,* respectively, were used to transform *Nicotiana* transiently (reporter gene YFP) and *Arabidopsis* stably (reporter gene GUS). Figure 5A illustrates that strong GUS signals are detected in all floral organs including sepals as well as in leaves for the promoter of *WSA206* (*MPF2-like-A* of *Withania*). These expression patterns are reminiscent of the native expression of *WSA206* in *Withania*. In the case of *WSB206* (*MPF2-like-B* of *Withania*), surprisingly, we detected very strong GUS signals in almost all the floral organs including sepals. Furthermore, when the GUS reporter was replaced with YFP and constructs were used to transiently transform *Nicotiana,* the YFP signals were equally strong for both the *WSA206* and *WSB206* promoters. This is an extraordinary result, which is in contrast with the native expression of *MPF2-like-B*. It is already mentioned that *MPF2-like-B* expression is not present in sepals. How *MPF2-like-B* promoters stimulate GUS expression in the heterologous systems to a stronger extent in flowers/sepals compared to the native background is an interesting question. One explanation could be that there are functional regulatory elements in the *MPF2-like-B* introns that are suppressing the effect of activator elements in the promoter responsible for floral expression including sepals. As most of the regulatory elements reported are enriched near the TSS [13], and as the large 1^st^ intron in the coding region of *MPF2-like* genes is located not very far from the TSS, we embarked on analyzing the effect of this intron on expression.

### Large 1^st^ Intron Suppresses the Expression of *MPF2-like-B* Genes of *Withania* (*WSB206*)

**Figure 6:**
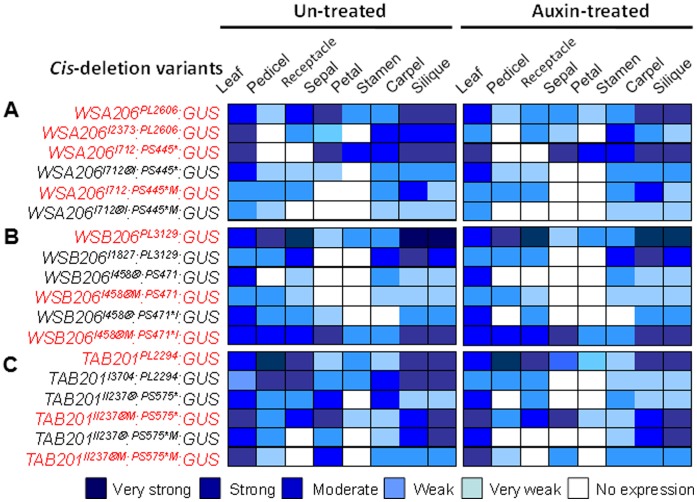
Auxin response factor element (ARE) suppresses the expression of *MPF2-like* genes in sepals activated by core promoter CArG-box. **ABC**) Heat map showing the qualitative tissue specific expression of GUS reporter in transgenic *Arabidopsis* driven under *MPF2-like* (*WSA206*, A; *WSB206*, B; *TAB201*, C) promoter and large 1^st^ intron regulatory elements is shown. The results of GUS patterns obtained from long promoter and complete large 1^st^ intron are included for comparison. Left panels (A–C) show the expression patterns observed without 10 µM auxin application, while GUS expression patterns of auxin-treated samples are shown in the right panels. The tissue specific expression of GUS reporter in 30 T3 homozygous transgenic lines is estimated. Legend is given at the bottom. The specifications of the constructs are described on the left side of heat map. Constructs lacking ARF site are marked as red letters. PL, promoter long; PI, promoter intermediate; PS, promoter short; I, *MPF2-like* large 1^st^ intron; * CArG-box; ⊗, ARE; M, mutated; *I, introduced CArG-box; I⊗, introduced ARE.

To investigate whether the complete large 1^st^ intron affects the expression of *MPF2-like* genes we used two types of constructs (Fig. S5). In the first type of constructs only promoter region (more than **−**2 kb upstream of the translational start site) was fused with the coding sequences of GUS and YFP reporter genes, respectively (*MPF2-like^PL^:GUS/YFP*). In contrast, in the second type of constructs the complete 1^st^ intron was additionally attached to the promoter sequence (*MPF2-like^I^:^PL^:GUS/YFP*). Figure 5ABC shows that YFP signal intensity is equal in almost all the *MPF2-like* promoter constructs except for the promoter of *MPF2-like-B* of *Withania* (*WSB206*) where it is slightly lower. However, YFP is barely detectable if the large 1^st^ intron is combined with the promoter for *WSB206*. In the other cases (*WSA206^I^:^PL^:YFP* and *TAB201^I^:^PL^:YFP*) no dramatic differences in YFP expression could be observed. These results were also confirmed by histological assay using GUS as a reporter gene (Fig. 6ABC). When the complete 1^st^ intron was attached to the *WSA206* promoter (*WSA206^I2373^:^PL2606^:GUS*) no noticeable change in the GUS expression pattern was observed and conversely, there was a dramatic reduction in GUS expression in case of combining *WSB206* 1^st^ intron and promoter (*WSB206^I1827^:^PL3129^:GUS*). This reduction in GUS expression was observed in most of the floral organs, particularly in sepals. This is congruent with the expression of *WSB206* in *Withania*.

From these data it can be deduced that the large 1^st^ intron suppresses the expression of *MPF2-like-B* genes of *Withania* (*WSB206*). Phylogenetic footprinting already indicated that an ARE is present in the 200 bp conserved region of all the *MPF2-like* large 1^st^ introns except *MPF2-like-A* of *Withania* (*WSA206*). The potential activity of an element can be missed if it is not analyzed in isolation. It will be interesting to study whether this ARE is responsible for suppressing the expression of *MPF2-like* genes, particularly the *MPF2-like-B* of *Withania*. For this purpose, reporter assay using site-directed mutagenesis and motif swapping of these elements was undertaken.

### ARF Binding Site Suppresses the Expression Activated by CArG-box

The effects of the ARE and the CArG-box on tissue specific expression of the GUS reporter gene in transgenic *Arabidopsis* were tested for promoters of *MPF2-like* genes (*WSA206*, *WSB206* and *TAB201*). For this purpose a total of 18 constructs - 6 constructs for each of the three promoters (*WSA206*, *WSB206* and *TAB201*) - were employed. Figure 6ABC (left panels) shows a heat map for qualitative expression observed for 30 plants for each construct. It depicts that in the case of the *WSA206* long promoter construct (*WSA206^PL2606^:GUS*) very strong GUS expression in both the leaf and floral organs including sepals was observed. Similarly, when combining the complete large 1^st^ intron with this promoter (*WSA206^I2373^:^PL2606^:GUS*), no deviation from this GUS expression pattern was visible. To investigate, whether the absence or presence of the mentioned ARE influences GUS expression, we introduced this site and used a short promoter containing the core-promoter CArG-box (*WSA206^I712^* ^∶*I*^ :^PS445*^:GUS). Interestingly, GUS signals became weak indicating that the ARE is suppressing the GUS expression in flowers, especially in sepal tissue. On the other hand, removal of the CArG-box (*WSA206^I712^* ^∶^ :^PS445*M^:GUS) strongly reduced the expression in sepals even if the ARE was not present. Introduction of an ARE (*WSA206^I712^* ^∶*I*^ :^PS445*M^:GUS), however, further reduced the expression below the detection level in sepals supporting a role of the ARE as a suppressor element with an antagonistic effect on expression compared to the activating CArG-box.

**Figure 7:**
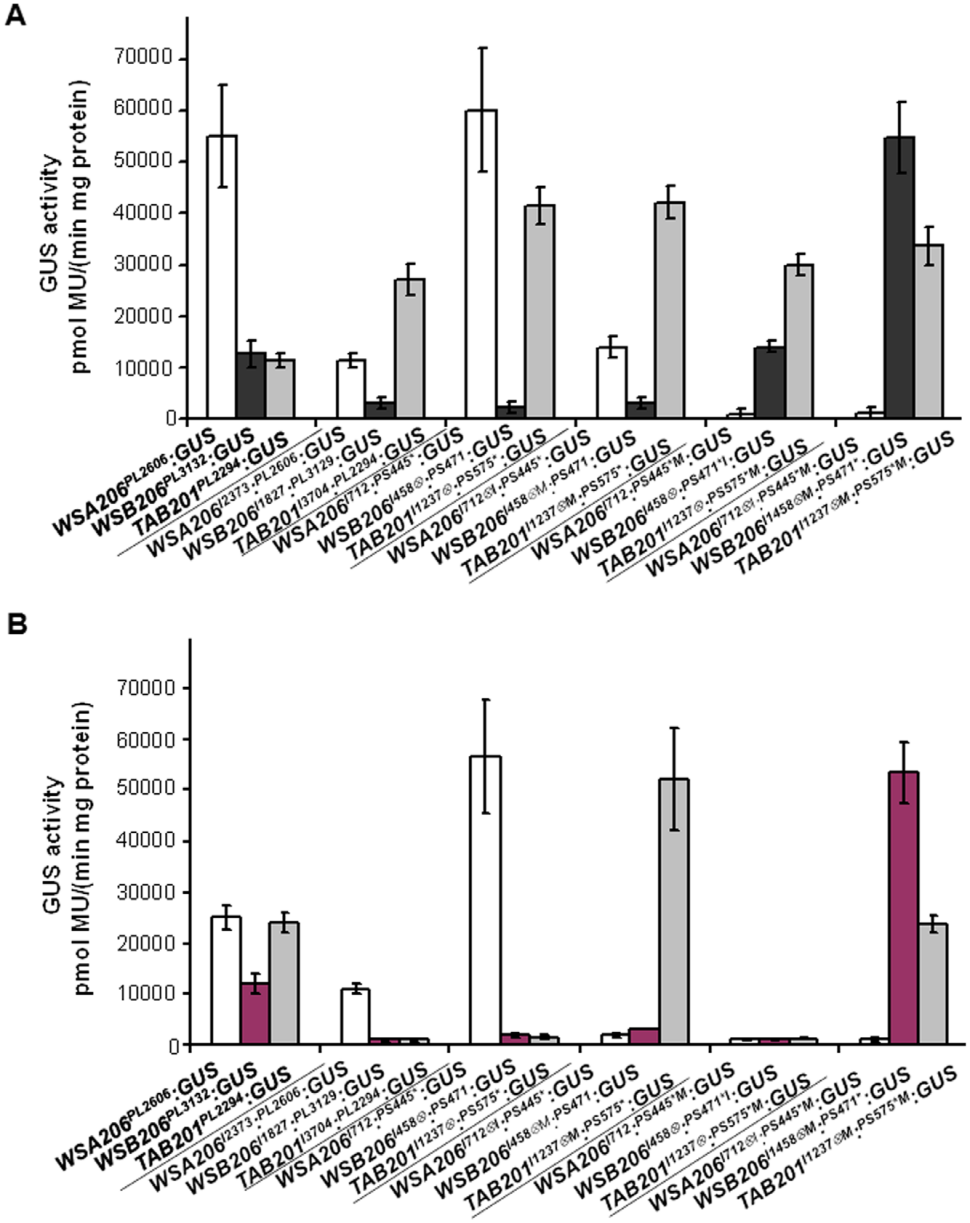
Interplay of auxin response factor element (ARE) and core promoter CArG-box with regard to expression of *MPF2-like* genes in sepals. **A**) Quantification of GUS enzymatic activity in the calyx of transgenic *Arabidopsis* by fluorometric method. Expression of the GUS gene is driven by *MPF2-like cis*-elements. **B**) Quantification of GUS enzymatic activity in the calyx of transgenic *Arabidopsis* after auxin spray by fluorometric method. The expression of the GUS gene is driven by *MPF2-like cis*-elements.

*WSB206 cis-*elements can help to improve our understanding of the interplay between the CArG-box and ARE. As already mentioned, the *WSB206* large 1^st^ intron contains a native ARE element but lacks the core promoter CArG-box. GUS expression was very strong in all the organs if only the long promoter was fused with the GUS cds (*WSB206^PL3129^:GUS*) but after attaching the complete 1^st^ intron (*WSB206^I1827^:^PL3129^:GUS*) there was a dramatic decrease in GUS signal intensity and expression in sepals became invisible. In order to further characterize the interaction between CArG-box and ARE, a short promoter without the CArG-box was combined with the intronic region (*WSB206^I458^* ^∶^ :^PS471*^:GUS). The resulting reduction in GUS expression in sepals could be a combined effect of the absence of the CArG-box and the presence of the ARE. However, if a CArG-box was introduced to the promoter and the ARE was mutated, GUS expression became very strong in all the floral organs including sepals. *TAB201 cis*-elements exhibited results not very different from *WSA206* with slight variations in GUS expression levels. These results are further supported by quantitative GUS expression analysis. For this purpose only calyx tissue was selected for determining the GUS activity by using fluorometric analysis. Figure 7A shows that GUS activity is similar to qualitative GUS expression for almost all the constructs for *WSA206*, *WSB206* and *TAB201 cis*-elements with subtle variations.

The interplay between ARE and CArG-box was also tested transiently in *Nicotiana* using the YFP reporter gene driven by *WSA206*, *WSB06*, *TAB201* and *MPF2 cis*-elements (Fig. S6). The results demonstrated that loss of the ARE relaxed constraints on the expression of the YFP reporter exerted by *MPF2-like* promoter or CArG-box elements.

### Application of Exogenous Auxin Suppresses the Expression of GUS Reporter Genes

Figure 6ABC (right panels) shows that transgenic *Arabidopsis* plants harboring only promoter-GUS constructs failed to respond to external auxin and exhibited no change in their GUS expression patterns. Furthermore, all the transgenic plants lacking an ARE in the construct including intronic sequence also showed no response to exogenous auxin. However, in cases where an ARE exists naturally or was introduced artificially, suppression of GUS signal intensity was observed. Hence, without auxin treatment the AREs mediate a suppression of GUS expression and this effect is more pronounced upon auxin treatment. In other words, an increase in auxin supply leads to an increased suppression of GUS expression. These results are supported by quantitative analysis of GUS activity in sepal tissues of the transgenic *Arabidopsis* plants after auxin treatment (Fig. 7B). This is a very interesting result, because it indicates an influence of auxin signaling on ICS development via mediating divergent expression of *MPF2-like* genes.

These cumulative data allow us to conclude that the ARE is an active suppressor of *MPF2-like* expression in sepals, which in turn is activated by the CArG-box. Thus reciprocal loss of core promoter CArG-box and intronic ARE in *MPF2-like-B* and *–A* genes, respectively, is responsible for expression divergence of *MPF2-like* genes and eventually influences ICS formation.

## Materials and Methods

### Plant Materials

A total of 13 accessions of Withania including 5 species (W. aristata, W. frutescens, W. riebeckii, W. coagulans and W. somnifera) and 1 accession each for Tubocapsicum anomalum, Vassobia breviflora, and Physalis floridana were grown in glasshouses of the Max-Planck-Institute for Plant Breeding Research (MPIPZ), Cologne, Germany, at 18–25°C (Table 1; [5]). Arabidopsis thaliana (ecotype Columbia) and Nicotiana benthamiana were grown under standard conditions. A few selected lines of transgenic Arabidopsis plants were also grown at the National Institute for Genomics and Advanced Biotechnology, Islamabad, Pakistan.

**Table 2:** Isolation of *MPF2-like* promoter and large 1^st^ intron sequences from five *Withania*, one *Tubocapsicum* and one species of *Vassobia*.

Sr. Nr.	Accession	Species	Gene	Promoter length (bp)	1^st^ intron length (bp)
1	W001	*W. aristata*	*WAA201*	2667	2373
2	W001	*W. aristata*	*WAB201*	3294	1827
3	W002	*W. coagulans*	*WCA202*	2609	2541
4	W002	*W. coagulans*	*WCB202*	2978	1865
5	W004	*W. riebeckii*	*WRA204*	2645	2621
6	W004	*W. riebeckii*	*WRB204*	3015	1873
7	W006	*W. somnifera*	*WSA206*	2606	2479
8	W006	*W. somnifera*	*WSB206*	3348	1945
9	W007	*W. somnifera*	*WSA207*	2645	2589
10	W007	*W. somnifera*	*WSB207*	3919	1870
11	W013	*W. frutescens*	*WFA213*	2645	2452
12	W013	*W. frutescens*	*WFB213*	2970	2001
13	T001	*T. anomalum*	*TAB201*	2308	3704
14	T002	*T. anomalum*	*TAB202*	2357	–
15	V001	*V. breviflora*	*V001_1*	2107	3386
16	V001	*V. breviflora*	*V001_2*	2056	–
17	P106^a^	*P. floridana*	*MPF2*	2238	2165
18	S032^a^	*S. tuberosum*	*STMADS16*	2588	2539

a Obtained from database; -, Could not be isolated.

### Measurement of Calyx Inflation and Scanning Electron Microscopy (SEM) of *Withania* and *Tubocapsicum* Sepals

In order to record the degree of inflation of the calyx after pollination, the length and width of flowering and fruiting calyces (10 each) of *W*. *somnifera*, *T*. *anomalum*, *P*. *floridana* and *V*. *breviflora* were measured using a Vernier scale. Data were statistically analyzed and inflation degree of the calyx was determined. Furthermore, morphological differences in the sepal cells of *W*. *somnifera* and *T*. *anomalum* were investigated by digital scanning electron microscopy (DSM940, Zeiss, Oberkochen, Germany; [4]).

### Expression Analysis

Expression of *MPF2-like* genes in *Withania*, *Tubocapsicum*, *Vassobia* and *Physalis* was investigated by real-time RT-PCR using the Bio-Rad iQ5 Real Time PCR Detection System. Total RNA extraction and first strand cDNA synthesis were performed [4]. A single forward primer flanking the conserved region coding for K- and C-domain was designed for *WSA206, WSB206*, *TAB201, V001* and *MPF2* genes was designed from the conserved regions. But the reverse primers were selected from the most polymorphic region of the cds. Reactions were carried out in a volume of 25 µl including 250 nmol/µl of gene-specific primers and 1XiQ SYBR Green Supermix solution (Bio-Rad). The reaction conditions were set at 95°C for 10 min to activate polymerase, followed by 40 cycles of 95°C for 30 sec, 60°C for 30 sec, and a final melting curve analysis of 60°C to 95°C. Only primer pairs showing 80–104% amplification efficiency were selected. The experiment was repeated three times for each of the biological as well as technical replicates. Relative expression of *MPF2-like* genes normalized with respect to 18 S *rRNA* was evaluated according to the Pfaffl method (Bio-Rad; [14]).

### Promoter and Large 1^st^ Intron Sequence Isolation

A total of 16 *MPF2-like* promoter sequences more than 2 kb in length (genomic sequence upstream of the MADS-box comprising 5′UTR) were isolated from various accessions of *Withania*, *Tubocapsicum* and *Vassobia* by RAGE (rapid amplification of genomic DNA ends) using the Universal Genome Walker Kit (Clontech; [5]). Their corresponding *MPF2-like* large 1^st^ intron sequences were isolated by PCR amplification by using primers designed to bind to promoter region and second exon of *MPF2-like* genes (Table 2) with Takara LA Taq polymerase.

### Phylogenetic Analyses and Transcription Factor Binding Site Identification

Maximum likelihood (ML) tree reconstruction was carried out with PAUP 4.0b10 [4], [15]. To evaluate the robustness of the tree structure, 1000 replicates of bootstrap searches were performed using maximum parsimony (MP) and maximum likelihood (ML) in PAUP and Bayesian posterior probability was calculated [16].

*MPF2-like cis*-regulatory sequences were assembled using MacVector™ 7.2.3 (Accelrys Inc.) gcg/Wisconsin Package University of Wisconsin) and AssemblyLIGN™ (Oxford Molecular Group Plc.) softwares. The ClustalW program in MacVector™ and mVista Shuffle-LAGAN were used to create multiple alignments (http://genome.lbl.gov/vista). *In silico* mapping of conserved transcription factor binding sites in *MPF2-like* promoters and large 1^st^ intron sequences was carried out using MULAN (Multiple-sequence local alignment; [17]) and MultiTF (Local multiple sequence alignments, transcription factor binding sites). Putative *cis*-regulatory elements were predicted using the TRANSFAC database of transcription factors (http://www.gene-regulation.com/pub/databases.html).

### Promoter:GUS/YFP and Intron:promoter:GUS/YFP Vector Construction

In order to investigate the effects of promoter and large 1^st^ intron elements on *MPF2-like* expression divergence, promoter:GUS/YFP and intron:promoter:GUS/YFP expression analyses were carried out in *Nicotiana* and *Arabidopsis* transiently and stably, respectively. Initially, a total of 16 constructs - 8 for each of the two reporters for four genes *WSA206*, *WSB206*, *TAB201*, and *MPF2* were generated. The 1^st^ construct type (e.g. *MPF2-like^PL^:GUS/YFP*) for each gene contains more than 2 kb of upstream genomic sequence. The 2^nd^ construct type (e.g. *MPF2-like^I^:^PL^:GUS/YFP*) harbors more than 2 kb of genomic region upstream of the translational start site fused with the complete 1^st^ intron (from 2 to 4 kb) containing both the 5′splice donor site of exon 1 (MADS-domain) and 3′ splice acceptor site of exon 2 (I-domain). This construct lacks the 180 bp of the MADS-box, and removing the ATG start codon prevents the expression of a truncated MPF2-like protein (containing only the MADS DNA binding domain). In order to decipher the role of the identified CArG-box close to the TSS, two special types of promoter constructs were generated in such a way that the promoter was considerably shortened and the naturally occurring CArG-box was included (MPF2-like-A^PS445*^:GUS/YFP), or mutated (MPF2-like-A^PS 445*M^:YFP) in *MPF2-like-A* of *Withania* and *MPF2-like-B* of *Tubocapsicum* (MPF2-like-A^PS575*^:GUS/YFP and MPF2-like-A^PS575*M^:GUS/YFP). However, for *MPF2-like-B* of *Withania* (*MPF2-like-B^PS471^:GUS/YFP*) that originally lacks the CArG-box, an artificial CArG-box was introduced (MPF2-like-B^PS471*I^:GUS/YFP).

In order to detect the interplay of ARE and CArG-box, 12 more constructs using GUS as a reporter - four for each of the three *MPF2-like* genes (*WSA206*, *WSB206* and *TAB201*) were designed. In these constructs the minimal promoter (short promoter containing CArG-box near the TSS) and only the region of the 1^st^ intron containing the ARE were included. In the first type of constructs, normal CArG-box and ARE sites were included but in the other three types, CArG-box and ARE were mutated or introduced through site-directed mutagenesis where present or absent, respectively. Motif swapping of CArG-box and ARE was also performed between *MPF2-like-A* and *-B cis*-elements. More details about the pursued scheme of promoter:reporter and intron:promoter:reporter construct making are given in figure S5.

Gateway (Invitrogen, http://www.invitrogen.com) expression vector pXCG-mYFP was used as a backbone to generate promoter-YFP and promoter-intron-YFP constructs whereas pGVT-bar was employed to make promoter-GUS and promoter-intron-GUS constructs. For making GUS constructs, both the *MPF2-like* regulatory elements and pGVT-bar vector were digested with *SmaI*, *XmaI*, *HindIII*, *SbfI* and *XbaI* restriction enzymes. After purification, ligation was done using T4 DNA ligase from NEB (New England Biolabs) at 15°C overnight. Sequenced plasmids containing inserts in the correct orientation and coding frame were transformed into the *A. tumefaciens* strain GV3101 (pMP90RK) for transient infiltration in *Nicotiana* and stable expression in *Arabidopsis*.

### Agroinfiltration in *Nicotiana* and YFP Signal Quantification

For transient infiltration, liquid YEB medium containing appropriate antibiotics was inoculated with *Agrobacterium* strain GV3101 (pMP90RK) harboring constructs with *MPF2-like cis*-elements fused with the YFP reporter along with constructs expressing the p19 protein [18]. These cultures were mixed before infiltration into *Nicotiana* leaves as described by [19]. The upper two healthy leaves were co-infiltrated using a 1 ml plastic syringe. Three days after infiltration, leaves of *N. benthamiana* were scanned using a Leica LCS SP2 AOBS^R^ Confocal Laser Scanning Microscope (CLSM) for YFP signal detection. At least 10 images showing YFP signals in the nuclei were selected randomly to quantify the luminescence with the Leica software LCS Lite (Leica Microsystems, Wetzlar, Germany). Promoter strength was determined as the relative intensity of YFP fluorescence in nuclei (mean of digital brightness values per nuclear area) for *MPF2-like cis*-element-YFP constructs in comparison with nuclear YFP fluorescence intensity of a 35 S promoter-YFP construct, which as the most intense signal, was set to 100%. The images were taken with the same hardware values for PMT gain, and offset and zoom.

### Transgenic Plant Production

Transformation of all the *MPF2-like* promoter: GUS and intron:promoter:GUS constructs along with the empty pGVT-bar vector into *Arabidopsis* was mediated by the GV3101 (pMP90RG) strain of *A. tumefaciens*. For plant transformation, a floral-dipping protocol was followed [20]. After seed germination, the transgenic plants were selected by spaying 0.15% Basta (Bayer Crop Science, Monheim) solution twice. T3 homozygous single copy transgenic lines were selected for reporter gene analysis.

### GUS Histochemical and Fluorometric Analysis

Histochemical GUS analysis was carried out to observe expression patterns of *MPF2-like* genes in different tissues of the transgenic *Arabidopsis* plants. Inflorescence, rosette leaves, siliques, small seedlings and entire plants of the transgenic *Arabidopsis* were stained with GUS staining solution [21]. More than 30 independent transgenic lines for each construct were examined for GUS activity. Calyx tissue was selected for GUS protein quantification. Quantitative assays were performed with 4-MUG as the substrate. Calyces of the transgenic plants before and after auxin spraying were harvested and homogenized in a 50 mM sodium phosphate lysis buffer (pH 7.0) containing 0.1% Triton X-100, 0.1% sodium lauroyl sarcosine, 10 mM EDTA and 10 mM β-mercaptoethanol. After centrifugation at 5000 rpm for 10 min, the GUS activity was evaluated using the resultant supernatant at 37°C in lysis buffer containing 1 mM 4-MUG. The reaction was terminated by adding 200 mM Na_2_CO_3_ to a final concentration of 160 mM. Fluorescence was quantified using a FLUOstar Galaxy multi-well plate reader with the excitation and the emission filters set at 365 nm and 455 nm, respectively. The GUS protein concentration was determined as described [22].

### DNA Sequencing

For all the PCR products, plasmids and constructs DNA sequences were determined by the MPIPZ DNA core facility on Applied Biosystems (Weiterstadt, Germany) Abi Prism 377, 3100 and 3730 sequencers using BigDye-terminator v3.1 chemistry.

**Figure 8:**
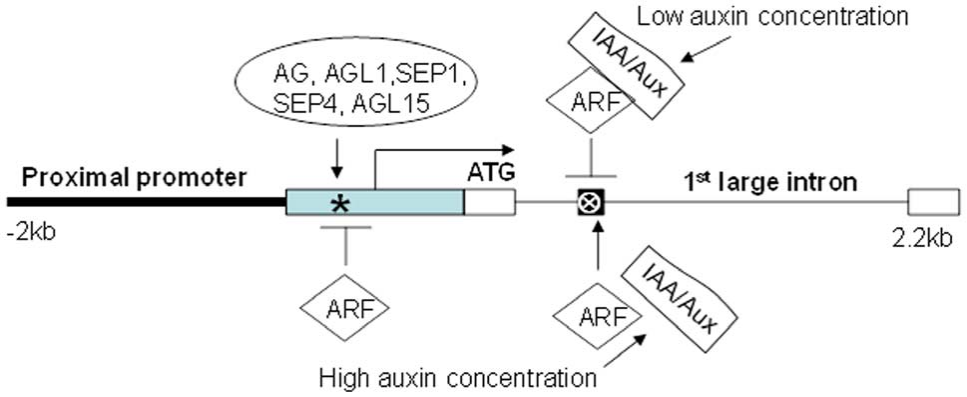
Auxin signaling as an input pathway of *MPF2-like* genes. Model depicting the interaction of CArG-box and ARF binding elements in *Arabidopsis* inferred through site-directed mutagenesis, motif swapping and auxin applications. Black bold solid line indicates proximal promoter and black solid line symbolizes the large 1^st^ intron in the coding region. Backward arrow marks the transcription start site. Arrow shows activation, while blocked line indicates repression. Deep sky blue box indicates promoter that allow constitutive expression in vegetative and floral tissues; *, core promoter CArG-box; Black solid box represent 200 bp highly conserved region in the large 1^st^ intron in the coding region; ⊗, ARF binding site; White empty solid box symbolizes exon.

## Discussion

### The Core Promoter CArG-motif is a Key Regulatory Element for *MPF2-like* Expression in the Calyx

MADS-domain proteins regulate expression of target genes by binding to CArG motifs in their promoter regions [23]–[25]. Most MADS-domain proteins prefer the so-called serum response element (SRE or SRF)-type CArG box, which has the consensus CC(A/T)_6_GG [26], [27]. Another closely related sequence motif is the MEF2-type CArG-box having the general consensus C(A/T)_8_G, but being usually more strictly defined as CTA(A/T)_4_TAG. However, plant MADS-domain proteins often show relatively broad DNA-binding preferences, recognizing SRF- and MEF2- as well as intermediate types of motifs [28].

Previously it was hypothesized that absence of two N10-like CArG-boxes in the promoter of *MPF2* of *Physalis* in comparison with *STMADS16* of *Solanum* accounts for the heterotopic expression of this gene in floral organs. This shift in vegetative to floral expression through *cis*-mutations ultimately leads to the formation of an inflated calyx [6]. However, this is merely an assumption that lacks functional evidence. In this study we have shown that different types of CArG-boxes (SRF and MEF2) frequently occur throughout 2 kb *MPF2-like* promoters and large 1^st^ introns (see “Results” section). Thus, presence of CArG-motifs alone is not sufficient to predict floral expression and targets of MADS-domain transcription factors [29]. Given the large number of MADS transcription factors and considering their divergent functions in plant development, it is imperative to elucidate their target-sequence specificity, for instance, what are the different types of CArG-boxes and what are the *cis*-regulatory positions that can be preferable sites for binding of MADS-domain interactors. Genome-wide analysis of *Arabidopsis* SEP3 revealed the highest enrichment of SRF- and intermediate types of CArG-boxes in the promoter region 1 kb upstream close to the TSS [13]. Hence it is suggested that recognition sites are not randomly distributed in the genome. Phylogenetic shadowing of *MPF2-like* short promoters identified an intermediate type of CArG-box with consensus sequence CCATAAAAAG in the close proximity of the TSS. The putative TSS of *MPF2-like* genes based on the sequence of the longest cDNA is **−**320 bp, **−**415 bp, **−**436 bp, **−**472 bp and **−**459 bp for *WSA206*, *WSB206*, *TAB201*, *MPF2* and *STMADS16*, respectively (Table 2). Hence, the position of our identified CArG-box in the regulatory region immediately upstream of the TSS in the core promoter of *MPF2-like* genes supports it to be functionally active.

**Figure 9:**
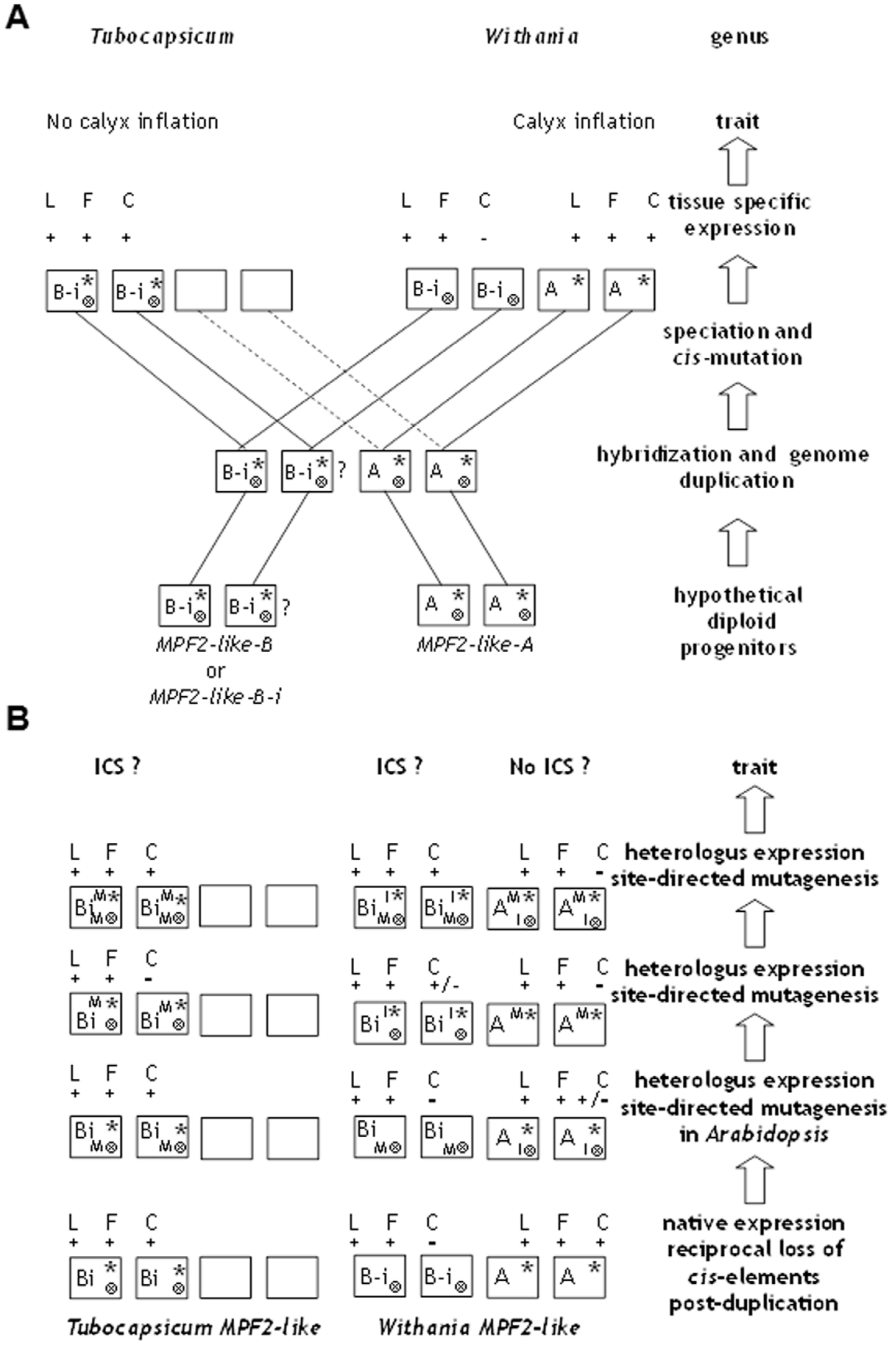
Contribution of *cis*-regulatory elements in the evolution of *MPF2-like* genes in *Withania* and *Tubocapsicum.* A ) The role of core promoter CArG-box and intronic ARF binding site in the evolutionary history of *MPF2-like* genes is shown. For details please see “Discussion” section. Wild type genes are shown as *MPF2-like-A* and *-B*. They are the hypothetical diploid progenitors. Upward empty arrow indicates stages in the evolutionary fate, expression and trait. Dotted line pointing to empty box represents gene loss; CArG-box is indicated as an asterisk (*). ARF binding site is marked as crossed circle (⊗). M, mutated; *I, introduced CArG-box; I⊗, introduced ARE. B-i is mutated version of *MPF2-like-B* gene, which is impaired in the ICS formation. L stands for expression in leaves, F for flower and C for calyx, respectively. +, expression is detectable; −, expression is absent. **B**) Model showing experimental *MPF2-like* expression analysis in *Arabidopsis* through site-directed mutagenesis and motif swapping using reporter genes. Upward empty arrow indicates experimental techniques. Dotted line pointing to empty box represents gene loss; CArG-box is indicated as an asterisk (*). ARF binding site is indicated as crossed circle (⊗). M, mutated; *I, introduced CArG-box; I⊗, introduced ARE. B-i is mutated version of *MPF2-like-B* gene, which is impaired in ICS formation. L stands for expression in leaves, F for flower and C for calyx, respectively. +, Expression is detectable; −, expression is absent; +/−, expression is doubtful.

The *MPF2-like* core promoter CArG-box is a potential binding site for the MADS-domain proteins AG, SEP1, SEP4, AGL3 and AGL15. MADS-domain proteins bind CArG-boxes as evident from electrophoretic mobility shift assays and chromatin immuno-precipitation experiments [30]. AG specifies floral meristem, carpel and stamen identity [31], [32]. The function of AGL2 and AGL3 is to define floral organ identity as well as to determine floral meristems [33], [34]. SEP4 has redundant functions in all the floral organs [33], while AGL15 plays a role in post-germinative development [35]. Hence the observed GUS expression patterns in floral tissue of transgenic *Arabidopsis* could be caused by MADS-domain proteins binding to the conserved CArG-box and up-regulating expression in floral organs. This is supported by recapitulation of *MPF2-like* transcript expression in floral organs of native plants. The core promoter CArG-box is found in all the *MPF2-like* promoters except *MPF2-like-B* of *Withania* (*WSB206*) and *STMADS16* of *Solanum*. Notably, both these genes are not expressed in sepals. Moreover, promoter-reporter experiments comprising site-directed mutagenesis and motif swapping confirmed that the core promoter CArG-box controls expression of *MPF2-like* genes in sepals. As expression of *MPF2-like* genes is essential for ICS formation, the core promoter CArG-box seems to be the key regulatory element for expression divergence of *MPF2-like* genes in the evolution of ICS.

### Auxin Signalling as an Input Pathway for *MPF2-like* Genes

Recently it was reported that ARF genes are not only targets of SEP3 MADS-box genes but also can be co-regulators [13]. ARF proteins recognize AREs in the intronic regions of target genes and modulate their expression. In *Arabidopsis* around 23 different types of ARFs have been identified so far. The amino acid contents in the variable middle region determine whether a particular ARF functions as a repressor or activator [36], [37]. Glutamine (Q) rich ARFs such as ARF5 and ARF7 activate transcription [12], [38]. All the ARFs bind to an auxin-response element (ARE), having the consensus sequence TGTCTC, in the regulatory regions of auxin response genes in an auxin dependent manner [39]. In this study, the type of binding site identified in *MPF2-like* 1^st^ intron is characteristic for an ARF with a Q-rich domain. Hence it might activate the expression. However, another class of proteins, Aux/IAA proteins, interact with ARFs and act as negative regulators of transcription [40]. Studying GUS expression patterns in *Arabidopsis* by site-directed mutagenesis and motif swapping of *MPF2-like cis*-elements (promoters with a CArG-box and intron with an ARE element) suggested that ARFs bind the ARE and suppress the expression of *MPF2-like* genes in flowers including sepals. The loss of the ARE in *MPF2-like-A* relaxes the expression constraints particularly on sepals. These results are supported by the native expression patterns. Interestingly, upon auxin treatment, *MPF2-like* expression is even more repressed. This repression may be either due to direct binding of ARFs to the ARE or there might exist an indirect pathway involving interaction of ARE with Aux/IAA. It is important to mention that *Arabidopsis* is evolutionarily distant from *Withania* but does possess *AGL24* and *SVP* as orthologs of Solanaceous *MPF2-like* genes. In the promoter and large 1^st^ intron of *AGL24* and *SVP* no ARF binding site exist (data not shown). Interestingly, there is no CArG-box near the transcriptional start site of *AGL24*, and in *SVP* it is present far away from the TSS. As *SVP* and *AGL24* lack core-promoter CArG-boxes, no strong expression in sepal is expected. Consequently, due to the absence of a CArG-box in *Arabidopsis* orthologs (*SVP* and *AGL24*) the roles of AREs in affecting flower expression cannot be explored. Furthermore in *Withania* and *Tubocapsicum,* cytokinin and auxin treatment does not alter the expression pattern at the transcript level (unpublished results).

The minima and maxima of endogenous auxin concentration influence flower morphogenesis [41]. The effects of auxin are thought to be dependent on its concentration; with high and low doses eliciting different responses [42], [43]. These interactions can be explained through a simple model. Figure 8 shows that *MPF2-like* expression activated via the core promoter CArG-box is suppressed through the ARE in the large 1^st^ intron. At lower concentration of auxin (endogenous in this case) ARFs might be bound by Aux/IAA proteins. This complex is unable to bind the ARE in the *MPF2-like* intron thereby allowing the transcription of *MPF2-like* genes to be activated by the core promoter CArG-box. An increase in auxin concentration (exogenous in this case) may cause the proteasome-mediated degradation of Aux/IAAs, as a result of which free ARF is able to bind the ARE in *MPF2-like* large 1^st^ intron. Hence, the ARF binding site acts as suppressor of *MPF2-like* expression particularly in sepals in contrast with the CArG-box, which is an activator element. This interaction is, however, not simple and might involve complex regulatory networks. The unravelling of the exact mechanism of transcriptional control through the ARE depends upon the availability of mutant ARF and *MPF2-like* lines. Nevertheless, this is the first time that a role of auxin in ICS development has been suggested at transcription level.

### Reciprocal Loss of *Cis*-regulatory Elements in the Evolution of *MPF2-like* Genes

According to the duplication-degeneration-complementation (DDC) model, sub-functionalization of duplicated genes predicts the reciprocal loss of *cis*-regulatory elements in each paralog by degenerative mutations such that paralog’s combined expression patterns recapitulate the expression patterns of the ancestor [44], [45]. A few examples that invoke the sub-functionalization of genes in plants exist but verification of reciprocal loss of *cis*-regulatory elements is lacking in plants [46]–[49]. There are a few examples of gene pairs such as *pax6a* and *pax6b* in vertebrates [50], which are consistent with the DDC model. The story of *hoxb5a* and *hoxb5b* is complex; there is no evidence for a simple loss of regulatory elements and the interactions between regulatory elements are not well understood [51]. Similarly, the sub-functionalization of *AGL6* and *AGL13* MADS-box genes is also based on the complex interactions of enhancer and silencer elements [46]. The evolution of *MPF2-like* genes in *Withania* seems to be an example of sub- and/or neofunctionalization according to the DDC model. The duplicated *MPF2-like-A* and *-B* genes show degenerative mutations in the promoter and large 1^st^ intron, respectively. The combined expression of the homologs and their contribution to function seem to be equivalent to the expression and functional contribution of unduplicated orthologs in closely related species.

Based on structural and functional characterization, the role of *cis*-regulatory elements in the evolutionary history of *MPF2-like* genes is summarized in Figure 9A. It is presumed that progenitors of *MPF2-like* genes contained a core promoter CArG-box (an asterisk marks CArG-box in *MPF2-like* promoters) near the TSS and an ARE (indicated by crossed circle) in the conserved block of the large 1^st^ intron. After, hybridization and allotetraploidization, both the lineages followed a normal speciation procedure and acquired their evolutionary fates. In the lineage *Withania*, *MPF2-like-A* retained the CArG-box and lost the ARF site. Consequently, *MPF2-like-A* of (*WSA206*) *Withania* was expressed in sepals due to the presence of CArG-box and was not repressed due to absence of an ARF binding site. Hence, *MPF2-like-A* could generate ICS in *Withania*. However, this gene was completely lost from the genome in the lineage *Tubocapsicum* (indicated by dotted line and empty box), wherefore there is no possibility of ICS formation in this genus. In the case of *MPF2-like-B* of *Withania* (*WSB206*) the CArG-box was lost. Furthermore, by retaining the ARE, *WSB206* failed to show any expression in sepals. Therefore, it has no capacity to generate ICS. On the other hand in *Tubocapsicum*, *MPF2-like-B* (*TAB201*) retained both the CArG-box and the ARF binding site. Consequently, it is expressed in the calyx due to relaxing of expression constraints but still impaired in ICS formation. No doubt, the expression of *MPF2-like* gene in the calyx is thought to contribute to the ICS, but availability of plant hormones cytokinins and gibberellins is also essential [4], [6]. Cytokinins facilitate the transport of MPF2 to the nuclei of the calyx cells thus promoting cell division resulting in smaller cells. These cells in the presence of gibberellins enlarge and ultimately form ICS [52]. Hence, the function of MPF2-like proteins is also regulated at the post-translational level. *Tubocapsicum* is sister to *Withania* but an “evolutionary loss mutant” of ICS. Therefore, *TAB201* is expressed in the flower but has lost the capacity to interact with hormones probably due to secondary mutations [4]. Interestingly removal of the CArG-box from *WSA206* and *TAB201* blocked expression of these genes in sepals, while its introduction in *WSB206* rescued the sepal expression (Fig. 9B). The ARE, on the other hand suppressed the expression of *WSB206* in flowers including sepals activated by CArG-box as revealed by site-directed mutagenesis and motif swapping. The expression in sepals is a pre-requisite for ICS formation but it is not the sole requirement. Hence the reciprocal loss of regulatory elements has a major impact on the expression evolvability or in other words sub-functionalization of *MPF2-like* genes.

The present study found evidence, that a protein similar to MPF2-like from *Vassobia* could be the progenitor of *MPF2-like* genes, particularly *MPF2-like-B*. Surprisingly, *V001* is expressed in the calyx but still does not feature ICS. How does this happen? Previously it was demonstrated that expression of *MPF2-like* genes in sepals is plesiomorphic and so might be the ICS trait [4]. This means that the ancestor of all Solanaceae might have exhibited ICS but later on due to secondary mutations (changes in protein structure precluding their interaction with hormones, which is important for the proliferation of ICS like structures), *Vassobia* would later have lost the ICS. As MPF2-like-B is subsumed to be contributed by Iochrominae, i.e. *Vassobia,* it would also not possess the ability to trigger ICS formation. The differences in protein structure for the respective genes support these speculations (Fig. S1). Moreover, the interactions of these proteins with hormones were altered and hence no ICS would be possible. This is supported by lack of calyx proliferation when hormones are applied to *Vassobia* flower buds [4], [53].

Recently it is demonstrated that expression evolvability may be subjected to selection [54]. Due to the co-dominant nature of *cis*-acting motif changes, selection acts on them rapidly unlike for coding sequences where loss-of-function changes are recessive [55]. In *Antirrhinum,* differences in flower color patterning that result from differences in the expression of the MYB transcription factors encoding *Rosea1*, *Rosea2*, and *Venosa* can affect pollinator attraction, and may have been under selection during speciation [56]. In a genome-scale investigation evidence for positive selection on putative transcription factor binding sites in human proximal promoters was found [57]. Nevertheless, MPF2-like-A from *Withania* and MPF2 from *Physalis* are under positive selection [5]. To link observed promoter sequence and expression divergence with positive selection, aiming to assess the contribution of regulatory changes in the evolution of ICS, it will be essential to perform a broad range of evolutionary and genome wide studies for these plant species. Nonetheless, the evolutionary history of *MPF2-like* genes allow us to conclude that expression divergence in *MPF2-like* genes due to reciprocal loss of *cis*-regulatory elements has added to genetic and/or phenotypic variations and enhanced the potential of natural selection for adaptive evolution. Moreover, this study highlights the contribution of allopolyploidy in plant evolution.

### Supporting Information

Figures S1, S2, S3, S4, S5 and S6 are available at *PLoS ONE* online (http://http://www.plosone.org). New promoter and intronic sequences have been submitted to NCBI database under the accession numbers –. Sequence data from this article can be found in the National Centre for Biotechnology Information (NCBI) under the following accession numbers: *MPF2* mRNA, (AY643734), *MPF2* (AY643735.2) *STMADS16* mRNA (AY643733) *STMADS16* (AY643736), *WSA206* mRNA (FM956486), *WSA206* (FM956482), *WSB206* mRNA (FM956487), *WSB206* (FM956483), *TAB201* mRNA (FM956485), *TAB201* (FM956484).

## Supporting Information

Figure S1 **ClustalW formatted multiple alignment of the amino acid sequences of WSA206, MPF2, V001, TAB201 and WSB206 proteins.** Blocked residues are conserved. Consensus sequence is also given at the bottom.
(TIF)

Figure S2 **ClustalW2 multiple alignment of the core promoter conserved block (B1) of** ***MPF2-like*** **promoter sequence (−150 bp to −650 bp).** Five conserved sequences stretches were identified and called *shadow 1* to *5* (*S1* to *S5*). Putative transcription start site is indicated. Dotted red box encloses the conserved CArG-box sequence.
(TIF)

Figure S3 **Conserved regulatory motifs in the promoter and large first intron of** ***MPF2-like*** **genes.** Arrow indicates the direction of transcription. Grey box is the MADS-box and two empty boxes represent the two exons in the upstream region. CArG-boxes occur frequently in the promoter and large 1^st^ intron. Red X represents the position of CC (A/T)7G CArG-box, which is a site for binding of AG, SEP1, SEP4, AGL1 and AGL15. Other types of CArG-boxes are shown as black X. Near the translational start site in the upstream region there are binding sites for Homeobox proteins such as ATHB1, ATHB5, AGP1 and P1. ARF binding site is shown as a crossed circle in the large 1^st^ intron. A rough scale and legend are also given.
(TIF)

Figure S4 **ClustalW multiple alignment of the conserved block (Block 3) of** ***MPF2-like*** **large 1^st^ intron sequence (1 bp to 500 bp).** Two conserved sequences stretches were identified and called *shadow 1* to *2* (*S1* to *S2*). Dotted red box indicates the conserved ARF binding site in *MPF2-like-B* large 1^st^ intron.
(TIF)

Figure S5 **Schematic of** ***MPF2-like*** **promoter: GUS/YFP and intron:promoter: GUS/YFP constructs making.** **A)** Shown are the 8 types of constructs using pGVT bar and pXCG-mYFP vectors containing GUS and YFP reporter genes as backbones, respectively. The red and white boxes represent the different lengths of *MPF2-like* promoters and introns. **B)** Twelve constructs using pGVT bar and pXCG-mYFP vectors containing GUS and YFP reporter genes as backbones are represented here to show the effects of CAArG-box and ARF binding site on expression of these genes. PS, promoter short; I, *MPF2-like* large 1^st^ intron; * CArG-box; ⊗, ARE; M, mutated; *I, introduced CArG-box; I⊗, introduced ARE. For details please see “Materials and Methods” section and “Table 2”.
(TIF)

Figure S6 **Graph shows the interplay of CArG-box and ARE using short promoter and large 1^st^ intron regions attached with YFP reporter.** A transient expression assay was performed using YFP reporter gene under the control of *MPF2-like* promoter and large 1^st^ intron. Three days after infiltration, leaves of *N. benthamiana* were scanned under Leica LCS SP2 AOBSR, Confocal Laser Scanning Microscope (CLSM) for YFP signal detection. At least 10 images selected randomly to quantify the luminescence with the Leica software LCS Lite. Promoter strength was determined as the relative intensity of YFP fluorescence of nuclear area of *MPF2-like* promoter YFP constructs in comparison with nuclear YFP fluorescence intensity of a 35 S promoter YFP construct. PS, promoter short; I, *MPF2-like* large 1st intron; * CArG-box; ⊗, ARE; M, mutated; *I, introduced CArG-box; I⊗, introduced ARE.
(TIF)
