# Supplementary figures and images for: Reciprocal Loss of CArG-Boxes and Auxin Response Elements Drives Expression Divergence of MPF2-Like MADS-Box Genes Controlling Calyx Inflation
Source: PLoS One. 2012 Aug 10;7(8):e42781. doi: 10.1371/journal.pone.0042781 (PMC3416756; doi:10.1371/journal.pone.0042781)

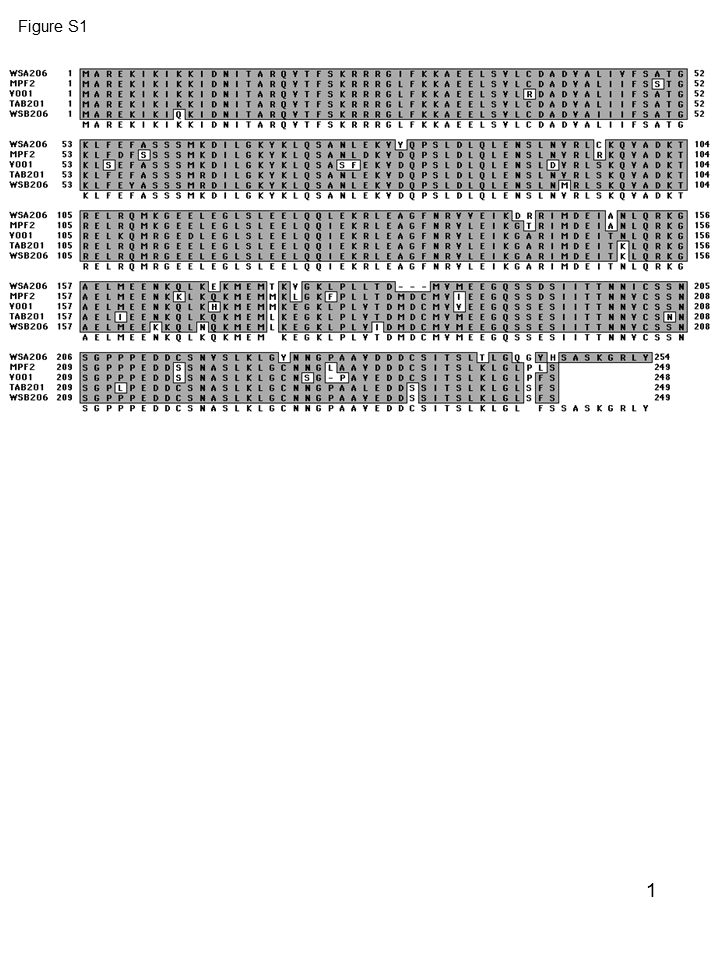

Supplement: Figure S1 — ClustalW formatted multiple alignment of the amino acid sequences of WSA206, MPF2, V001, TAB201 and WSB206 proteins. Blocked residues are conserved. Consensus sequence is also given at the bottom. (TIF) [file pone.0042781.s001.tif]

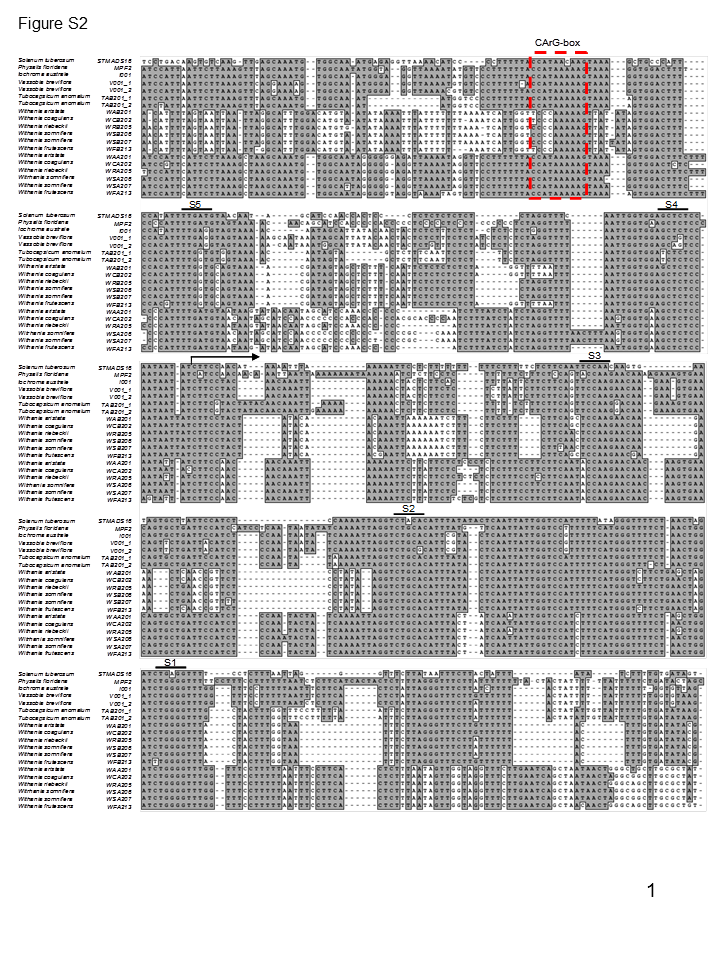

Supplement: Figure S2 — ClustalW2 multiple alignment of the core promoter conserved block (B1) of MPF2-like promoter sequence (−150 bp to −650 bp). Five conserved sequences stretches were identified and called shadow 1 to 5 (S1 to S5). Putative transcription start site is indicated. Dotted red box encloses the conserved CArG-box sequence. (TIF) [file pone.0042781.s002.tif]

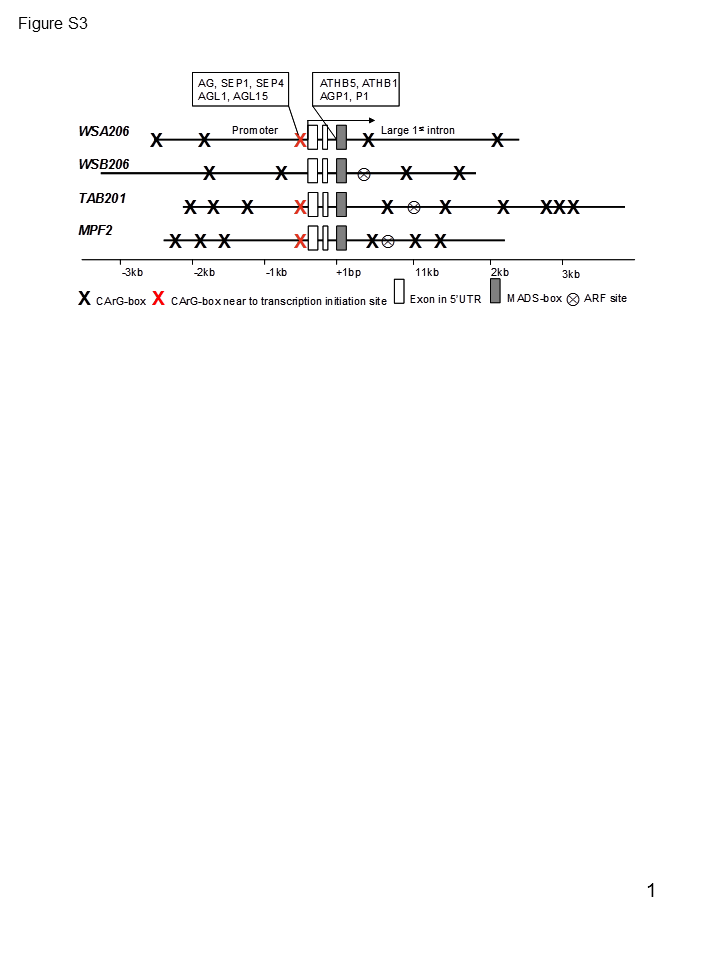

Supplement: Figure S3 — Conserved regulatory motifs in the promoter and large first intron of MPF2-like genes. Arrow indicates the direction of transcription. Grey box is the MADS-box and two empty boxes represent the two exons in the upstream region. CArG-boxes occur frequently in the promoter and large 1st intron. Red X represents the position of CC (A/T)7G CArG-box, which is a site for binding of AG, SEP1, SEP4, AGL1 and AGL15. Other types of CArG-boxes are shown as black X. Near the translational start site in the upstream region there are binding sites for Homeobox proteins such as ATHB1, ATHB5, AGP1 and P1. ARF binding site is shown as a crossed circle in the large 1st intron. A rough scale and legend are also given. (TIF) [file pone.0042781.s003.tif]

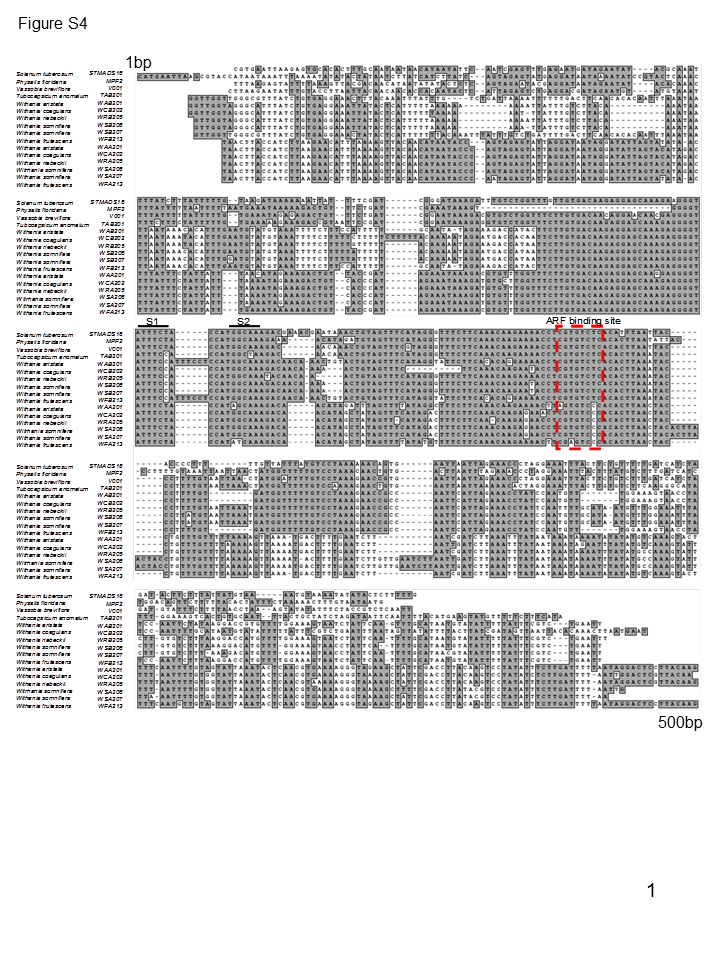

Supplement: Figure S4 — ClustalW multiple alignment of the conserved block (Block 3) of MPF2-like large 1st intron sequence (1 bp to 500 bp). Two conserved sequences stretches were identified and called shadow 1 to 2 (S1 to S2). Dotted red box indicates the conserved ARF binding site in MPF2-like-B large 1st intron. (TIF) [file pone.0042781.s004.tif]

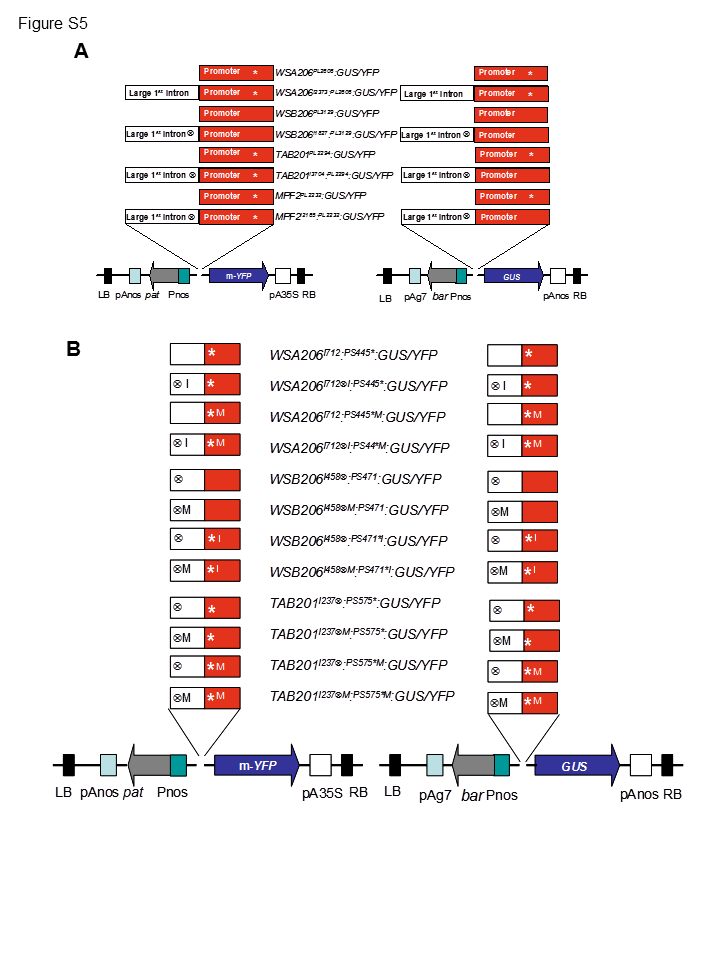

Supplement: Figure S5 — Schematic of MPF2-like promoter: GUS/YFP and intron:promoter: GUS/YFP constructs making. A) Shown are the 8 types of constructs using pGVT bar and pXCG-mYFP vectors containing GUS and YFP reporter genes as backbones, respectively. The red and white boxes represent the different lengths of MPF2-like promoters and introns. B) Twelve constructs using pGVT bar and pXCG-mYFP vectors containing GUS and YFP reporter genes as backbones are represented here to show the effects of CAArG-box and ARF binding site on expression of these genes. PS, promoter short; I, MPF2-like large 1st intron; * CArG-box; ⊗, ARE; M, mutated; *I, introduced CArG-box; I⊗, introduced ARE. For details please see “Materials and Methods” section and “Table 2”. (TIF) [file pone.0042781.s005.tif]

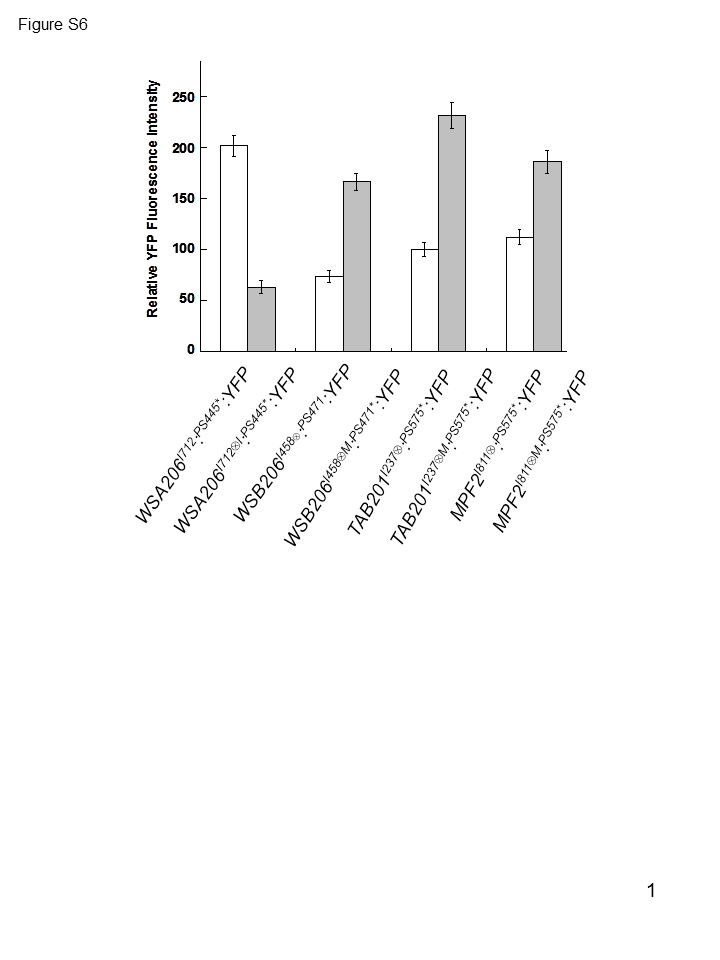

Supplement: Figure S6 — Graph shows the interplay of CArG-box and ARE using short promoter and large 1st intron regions attached with YFP reporter. A transient expression assay was performed using YFP reporter gene under the control of MPF2-like promoter and large 1st intron. Three days after infiltration, leaves of N. benthamiana were scanned under Leica LCS SP2 AOBSR, Confocal Laser Scanning Microscope (CLSM) for YFP signal detection. At least 10 images selected randomly to quantify the luminescence with the Leica software LCS Lite. Promoter strength was determined as the relative intensity of YFP fluorescence of nuclear area of MPF2-like promoter YFP constructs in comparison with nuclear YFP fluorescence intensity of a 35 S promoter YFP construct. PS, promoter short; I, MPF2-like large 1st intron; * CArG-box; ⊗, ARE; M, mutated; *I, introduced CArG-box; I⊗, introduced ARE. (TIF) [file pone.0042781.s006.tif]
